# Metabolic and Gut Microbiome Responses to Paraquat Exposure in *Apis mellifera* Under Laboratory Conditions

**DOI:** 10.3390/insects17060632

**Published:** 2026-06-15

**Authors:** Natasha Mills, Nantana Mills, Patcharin Phokasem, Giatgong Konguthaithip, Rujipas Yongsawas, Chanon Saksunwiriya, Chainarong Sinpoo, Sahutchai Inwongwan, Sasiprapa Krongdang, Ji-Ho Lee, Churdsak Jaikang, Terd Disayathanoowat

**Affiliations:** 1 Department of Biology, Faculty of Science, Chiang Mai University, Chiang Mai 50200, Thailand; natasha_mills@cmu.ac.th (N.M.); nantana_mills@cmu.ac.th (N.M.); patcharin.ph@cmu.ac.th (P.P.); r.yongsawas@gmail.com (R.Y.); chanon_viriya@hotmail.com (C.S.); chainarong.s@cmu.ac.th (C.S.); sahutchai.inwongwan@cmu.ac.th (S.I.); 2 Research Center of Deep Technology in Beekeeping and Bee Products for Sustainable Development Goals (SMART BEE SDGs), Chiang Mai University, Chiang Mai 50200, Thailand; 3 Metabolomics Research Group for Forensic Medicine and Toxicology, Department of Forensic Medicine, Faculty of Medicine, Chiang Mai University, Chiang Mai 50200, Thailand; kongkiat.shang@gmail.com; 4 Office of Research Administration, Chiang Mai University, Chiang Mai 50200, Thailand; 5 Faculty of Science and Social Sciences, Burapha University, Sa Kaeo Campus, Sa Kaeo 27160, Thailand; sasiprapa.kr@buu.ac.th; 6 School of Natural Resources and Environmental Science, Department of Biological Environment, Kangwon National University, Chuncheon 24341, Gangwon, Republic of Korea; micai1@naver.com

**Keywords:** 16S rRNA amplicon sequencing, central carbon metabolism, energy metabolism, gut bacterial community, herbicide, metabolomic, Western honeybee

## Abstract

Paraquat is a widely used herbicide known to adversely affect honeybee physiology; however, its impact on gut-associated biological processes remains poorly understood. Therefore, intricate effects of paraquat on honeybees highlight the need for a comprehensive understanding of its specific biological consequences. This study aimed to evaluate the gut microbiome composition and metabolomic profiles of honeybees following oral exposure to sublethal (LD_25_) and toxic (LD_50_) doses of paraquat, assessed 48 h post-exposure to represent acute exposure. Survival rate and sucrose consumption were monitored to assess toxicity. Honeybee gut samples were analyzed using 16S rRNA gene amplicon sequencing and metabolomic approaches. Both paraquat treatments resulted in increased mortality (*p* < 0.0001) and reduced sucrose consumption (*p* = 0.00503). In contrast, gut bacterial community composition remained largely unchanged (*p* > 0.05). Metabolomic analysis revealed dose-dependent alterations, particularly in metabolites associated with carbohydrate and energy metabolism, including oxaloacetic acid and pyruvic acid. This study provides the first integration of 16S rRNA gene amplicon sequencing and metabolomic analyses to evaluate the effects of paraquat exposure in honeybees. The findings indicate that paraquat-induced stress primarily exhibits as host metabolic reprogramming rather than changes in microbiome composition. These results provide insights into the mechanisms underlying honeybee responses to herbicide stress and may contribute to the development of strategies for honeybee protection.

## Simple Summary

The Western honeybee, *Apis mellifera*, plays a vital role in ecosystems and in the commercial honey industry. While managed honeybee colonies are generally maintained through beekeeping practices, increased exposure to agricultural chemicals, such as herbicides, can contribute to honeybee population declines and reduce the number of colonies in beekeeping operations as well as decline other wild pollinators. Paraquat, an herbicide, is still widely used in some regions, but its effects on honeybees’ gut-associated biological processes in honeybees remain incompletely understood. To better understand how paraquat affects honeybee health, we investigated both gut microbiome composition and host metabolic responses following paraquat exposure. Honeybees were exposed to sublethal and toxic doses of paraquat and compared with unexposed controls. Under the conditions tested, paraquat exposure did not significantly alter gut microbiome composition. In contrast, it affected honeybee metabolism, including several cellular pathways and key metabolite compounds. Overall, our findings indicate that paraquat exposure is associated with increased and altered metabolic profiles, which can harm honeybee health, highlighting potential risks of herbicide use and the need for careful management to protect both wild pollinators and honeybees.

## 1. Introduction

Pollination is essential for ecosystems, maintaining biodiversity and contributing to the reproduction of global plants and crops, with 70% of global food crops depending on insect pollination for productivity [1,2,3]. Among insect pollinators, the Western honeybee (*Apis mellifera*) is considered one of the most effective pollinators, contributing to high-frequency plant-pollinator interactions and supporting the pollination of both agricultural and wild plants. While *A. mellifera* serves as a primary pollinator in industrial agricultural systems and enhances crop yield and quality, its function within a diverse community of wild pollinators remains essential for maintaining natural ecosystems and collectively enhancing crop productivity [3,4,5,6,7,8]. Beyond its ecological importance, *A. mellifera* also sustains significant economic value through various honeybee products and other bioactive products with nutritional, medicinal, and cosmetic applications [3,9,10,11]. However, *A. mellifera* currently faces serious challenges due to population decline and colony losses.

Honeybees in the United States experienced a 62% and 39.0% colony loss during 2021–2022, with annual losses of about 40.9% [12]. Similarly, in Australia and some European countries, approximately 40% of honeybee colonies have been lost [13], while in Latin America, annual colony losses reach about 30.4% [14]. These declines and colony losses result from both biotic stresses, including climate change, ecosystem shifts, deforestation, and pathogen and parasite infections, as well as abiotic stresses, such as exposure to agricultural chemicals, including pesticides, insecticides, fungicides, and herbicides [5,15,16,17]. Among these, abiotic stress has become a serious concern, as the global use of agricultural chemicals has increased markedly in recent decades due to agricultural expansion and population growth, resulting in frequent honeybee exposure [12,18,19].

Herbicides are the most frequently used, accounting for about 50% of all pesticides, and are used extensively in Asia [19,20,21,22]. Although primarily designed to target plants, their toxicological mode of action can affect other nontarget organisms. Herbicide residues and sublethal exposures may adversely affect insects over time, and elevated direct exposure to toxic compounds or chemicals in herbicides may induce oxidative stress in insects [21,22]. Among herbicides, paraquat is one of the most widely applied [23]. Honeybees are often exposed to agrochemicals during spray drift, foraging, visiting treated plants, and collecting nectar and pollen contaminated with the chemicals, particularly when high concentrations are applied during the blooming period [12,15,16,24,25,26,27]. Furthermore, honeybees can transport contaminated nectar and pollen back to their hives, where chemical residues have been detected, which can affect bee survival and contribute to colony losses [26,28].

Paraquat (1,1′-dimethyl-4,4′-bipyridylium dichloride) is a widely used herbicide, particularly for weed and grass control in agricultural fields, and can induce oxidative stress by generating reactive oxygen species (ROS) [22,23,29,30]. Despite bans in 67 countries, including Thailand [31], paraquat continues to be detected in the environment due to residual contamination, where it can persist in areas where pollinators forage, and illegal use and sales persist because of its low cost and high efficacy [23,30]. Paraquat exposure has been demonstrated to accumulate and exert effects across a wide range of non-target organisms, including mammals, birds, fish, frogs, crabs, and invertebrates [23,29,32].

Numerous studies have demonstrated the effects of paraquat on insects, such as reduced oviposition and lifespan in *Diaeretiella rapae* M’Intosh [33], induction of oxidative stress, high mortality, and impaired movement in *Drosophila melanogaster* (fruit fly), *Temnothorax rugatulus* (Myrmicine ants), and *Atta colombica* and *Acromyrmex echinatior* (leaf-cutting ants) [34,35,36,37]. However, paraquat affects not only adult insects but also their larvae by impairing growth, development, and survival in *Tenebrio molitor* (yellow mealworm) and *A. mellifera* larvae [38,39]. Regarding adult honeybees, the Western honeybee (*A. mellifera* L.), African honeybee (*A. mellifera scutellata* Lepeletier), and Carniolan honeybee (*A. mellifera* carnica) were studied and showed that paraquat was linked to increased mortality and oxidative stress, associated with pathogen infection, impaired detoxification and reduced sucrose consumption after paraquat exposure [16,40,41]. A recent study from Phokasem et al. (2024) [42], further emphasized paraquat’s impact on *A. mellifera* survival, showing increased mortality over time, along with the altered expression of genes involved in detoxification, oxidative stress response, and immune regulation.

However, paraquat’s effects have been studied primarily at the physiological and survival levels, while its impact on the gut microbiome of *A. mellifera* remains unexplored, despite the critical role of the honeybee gut microbiome in supporting nutrition, metabolism, and immune defense, which collectively contribute to colony health [43,44]. By contrast, several studies have demonstrated the impact of glyphosate, another herbicide, on the gut microbiome of honeybees and bumblebees [45,46,47,48,49,50,51,52]. Additionally, there remains a gap in understanding the host’s biological processes and gut-associated metabolomic responses under paraquat stress. In particular, the effects of paraquat on gut microbiota and metabolic processes in *A. mellifera* have not been thoroughly investigated.

Understanding these complex interactions requires more than traditional physiological assays; integrated ‘omics’ approaches are essential to capture the interactions fully. Therefore, to address these gaps, integrated 16S rRNA gene amplicon sequencing and metabolomic approaches can provide detailed insights into both microbiome alterations and host responses in paraquat-exposed honeybees. The objectives of this study are to compare the gut microbiome composition and metabolomic profiles of non-exposed honeybees (*A. mellifera*) with those exposed to paraquat at sublethal (LD_25_) and toxic (LD_50_) doses, to identify differences and assess their impacts on honeybee health.

## 2. Materials and Methods

### 2.1. Chemicals

Herbicide treatment was conducted using paraquat dichloride (99.9% purity; LGC-Dr. Ehrenstorfer, Augsburg, Germany). A stock solution of paraquat was prepared by dissolving 4 mg of paraquat in deionized water to obtain a final concentration of 4 mg/mL, and then the paraquat stock solution was shielded from light using aluminum foil and stored at 4 °C until use. In the experiment, the feeding solution of paraquat was prepared by diluting the paraquat stock with sterile 50% (*w*/*v*) sucrose solution. For the metabolomic analysis, chemicals were obtained from Sigma-Aldrich (St. Louis, MO, USA), including chloroform and 0.1 M trimethylsilyl propanoic acid (98% purity; TSP) in 0.6 mL deuterium oxide (99.9% purity; D_2_O) solution. These chemicals were used for sample preparation and extraction.

### 2.2. Experimental Honeybees and Sampling

Frames containing a capped brood of *A. mellifera* were randomly collected from two apiaries in Chiang Mai, Thailand. Three colonies were sampled from each apiary, and one capped-brood frame was collected from each colony [53]. The capped brood frames were selected from healthy colonies with no signs of infection and no prior exposure to chemicals or miticides [53]. The capped brood frames were transferred to the laboratory and placed in an incubator maintained at 34 ± 1 °C with 70 ± 5% relative humidity. Newly emerged worker honeybees were allowed to emerge naturally on their original frames to ensure acquisition of the gut microbiome. After 24 h, newly emerged worker honeybees were randomly collected, pooled across all sampled colonies, and placed into hoarding cages containing 30 honeybees per cage [53]. This approach minimized potential confounding effects arising from colony origin and genetic variation, thereby allowing the treatment effects to be assessed more accurately. The bee hoarding cages were constructed from plastic cup-shaped containers with a diameter of 7 cm and an internal volume of 153 cm^3^. Each cage was equipped with a modified feeder, constructed from a sterile 3 mL syringe, which was vertically mounted at the center of the cage’s upper side [53]. All honeybees were then placed in a controlled incubator at 34 ± 1 °C with 70 ± 5% relative humidity and provided with a sterile 50% (*w*/*v*) sucrose solution *ad libitum* until the oral toxicity assay [53].

### 2.3. Oral Toxicity Assay

**Figure 1:**
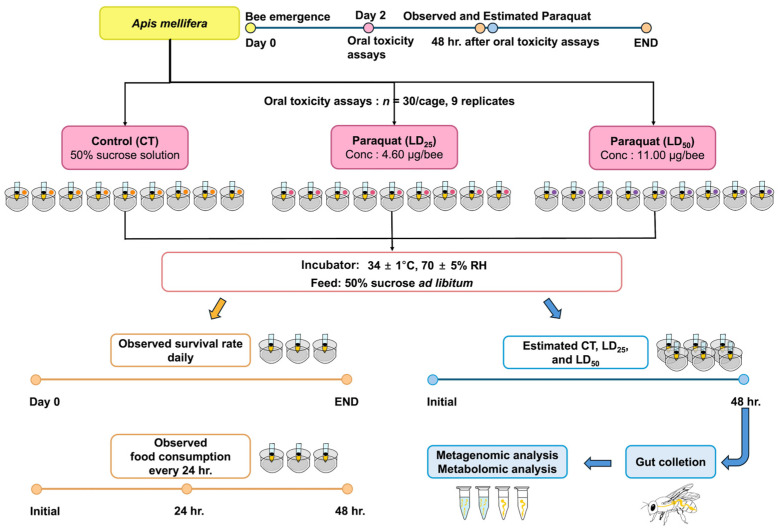
Schematic of experimental design for exposure and analysis of this study.

For the oral toxicity assay, 48-h-old worker honeybees were allocated to three groups, each comprising nine replicates (cages) with 30 honeybees per cage. The selection of 48-h-old worker honeybees for paraquat exposure followed the OECD Oral Toxicity Test protocol, which requires administration of the toxicant after a 2-day acclimation period [54]. Each group received a different solution and a different dose of paraquat using an individual feeding method (Figure 1). Initially, all honeybees were starved for 2 h prior to paraquat exposure [55]. Subsequently, each honeybee was individually fed 5 µL of solution using a micropipette, with each bee receiving a single feeding [56]. The control group (CT) received a sterile 50% (*w*/*v*) sucrose solution, while the paraquat treatment groups received two different doses of paraquat, consisting of sublethal (LD_25_) and toxic (LD_50_) doses. Honeybees in the sublethal dose group (LD_25_) were exposed to paraquat at a concentration of 4.6 µg/bee, while those in the toxic dose group (LD_50_) were exposed to paraquat at a concentration of 11 µg/bee [42]. After the assay, all honeybees were kept in a controlled incubator at 34 ± 1 °C with 70 ± 5% relative humidity and provided with a sterile 50% (*w*/*v*) sucrose solution *ad libitum* [53].

### 2.4. Paraquat Treatment with Survival Rate and Food Consumption of Apis mellifera

For survival and food consumption observations, three replicates (cages) from each group were monitored (Figure 1). To assess the survival rate, the honeybee mortality was first recorded at 24 h post-exposure to paraquat and subsequently recorded every 24 h until all honeybees died. The number of dead honeybees was counted and removed daily for hygiene reasons. Similarly, for food consumption monitoring, sucrose consumption was first recorded at 24 h post-exposure to paraquat. The initial sucrose weight was recorded, followed by measurements of the remaining sucrose weight every 24 h until 48 h. The assessment of the survival rate and food consumption of *A. mellifera* was modified from Almasri et al. (2022) [57].

### 2.5. Gut Collection

Honeybee samples at 48 h post-exposure to paraquat were used for gut sample collection. This time point was selected based on previous studies by Phokasem et al. (2024) [42], representing acute exposure conditions. For sterility, a surface sterilization technique was performed by immersing whole honeybees in 3% sodium hypochlorite for 1 min, followed by 70% ethanol for 3 min, and rinsing three times with sterile water for 1 min each [58]. The bees were then dried using sterile tissue paper. For gut dissection, honeybees were placed on sterile plastic petri dishes, and sterile forceps were used to collect the gut by grasping the stinger and carefully pulling out the entire gut. This method was modified from Carreck et al. (2013) [59]. The dissected gut samples were stored at −20 °C for further analysis.

### 2.6. 16S rRNA Gene Amplicon Sequencing Analysis

#### 2.6.1. Sample Preparation and Genomic DNA Extraction

The gut samples were collected, and six replicates (cages) were used for each group, with each replicate consisting of a pooled sample of three gut samples. Pooling was performed to obtain a reliable and comprehensive representation of the gut bacteria in honeybees. The pooled gut samples were lysed by grinding the gut tissue with DNA/RNA Shield™ (ZYMO Research, Freiburg im Breisgau, Germany) using sterile plastic pestles. The homogenized samples were then transferred to ZR BashingBead™ lysis tubes (ZYMO Research, Freiburg im Breisgau, Germany) and further lysed for 30 min using a bead beater homogenizer (Disruptor Genie, Scientific Industries, Bohemia, NY, USA).

For genomic DNA extraction, DNA was extracted using the ZymoBIOMICS^TM^ DNA Miniprep Kit (ZYMO Research, Freiburg im Breisgau, Germany) according to the manufacturer’s protocol. The DNA concentration, quantification, and purity were measured using a NanoDrop UV–Vis spectrophotometer (NanoDrop™ Lite Plus Microvolume Spectrophotometer, Thermo Fisher Scientific, Waltham, MA, USA).

#### 2.6.2. Bacterial 16S rRNA Gene Amplicon

The extracted DNA samples were sent for library construction and sequencing using the Next-Generation Sequencing (NGS) method via the Illumina paired-end MiSeq platform through Macrogen, Inc. (Seoul, Republic of Korea). The 16S rRNA fragment was amplified using bacteria primer sets: the forward primer was 341F (5′-CCTACGGGNGGCWGCAG-3′), and the reverse primer was 805R (5′-GACTACHVGGGTATCTAATCC-3′) [60]. The DNA reads were analyzed, processed, and classified using QIIME2 (Quantitative Insights Into Microbial Ecology 2) version 2024.10 [61], where the raw DNA reads in FASTQ format were trimmed to remove primers and adapters, followed by data filtering using the DADA2 (Divisive Amplicon Denoising Algorithm) package to remove low-quality sequences [62]. Forward and reverse sequences were then merged, and singletons were removed, as they may represent sequencing errors. A rarefaction curve and rarefy were generated to ensure sequence stabilization and normalization per sample, respectively, and to reduce the bias associated with uneven sampling effort that can affect microbial diversity analysis [63,64]. The obtained sequences were taxonomically classified using the SILVA 138 database, with mitochondrial and chloroplast sequences removed to eliminate the false identification of bacteria [65]. Subsequently, four types of analyses were performed on the received bacterial data, consisting of the gut bacterial community composition, bacterial microbiome diversity, correlations within the bacterial microbiome, and the functional properties of gut bacteria.

#### 2.6.3. Data Processing and Statistical Analysis

All data were statistically analyzed and visualized using the R environment (version 4.4.1). For survival analysis, the R packages “survival” and “survminer” were used with the Kaplan–Meier survival probability model to visualize survival trends across control and treatment groups. A log-rank (Chi-square, *X*^2^) test was conducted to assess the overall survival differences among all groups. In addition, pairwise survival comparisons were performed using Bonferroni correction [66]. The survival rate curve figure was also generated using the same packages.

For food consumption analysis, the R packages “dplyr” and “multcompView” were used to perform statistical testing, and “ggplot2” was used to generate the figure [67,68,69]. Sucrose consumption was visualized using box plots, statistical analysis was conducted using one-way ANOVA with Tukey’s correction for multiple comparisons, and *p* < 0.05 was considered statistically significant.

The gut bacterial community was analyzed based on relative abundance and visualized using the “ggplot2” package in R [67]. To assess the differences in the bacterial composition between the control and treatment groups, the “MaAsLin2” package in R was employed [70]. Statistical significance was determined using multiple comparisons, with *p* < 0.05 considered significant. The analysis also reported coefficient values, standard errors, and false discovery rates (FDR) for each comparison.

Bacterial microbiome diversity was analyzed using alpha and beta diversity [71,72]. Alpha diversity was assessed using three metrics including Shannon, Simpson, and Observe richness. The analysis was performed using the “vegan” package in R to calculate all metrics [73], and “ggplot2” package was used to generate box plot visualizations [67]. Statistical comparisons were conducted using the Kruskal–Wallis test, with *p* < 0.05 considered statistically significant [74]. Beta diversity was visualized using non-metric multidimensional scaling (NMDS) via the “vegan” package in R, and statistical analysis was based on Bray–Curtis dissimilarity, with differences were evaluated using PERMANOVA (*p* < 0.05) [73,75]. To generate the NMDS figure, the R package “ggplot2” was used.

The correlations within the bacterial microbiome were analyzed using network analysis. The analysis was performed using the “vegan” and “Hmisc” packages in R to assess pairwise correlation, with *p* < 0.05 considered statistically significant and a correlation coefficient (*r*) greater than 0.7 representing a strong correlation [60,76]. For the figure, the network visualization was generated using Gephi (version 0.10.1) with the Fruchterman Regingold model [77].

The functional properties of gut bacteria were analyzed using PICRUSt2 (Phylogenetic Investigation of Communities by Reconstruction of Unobserved States, version 2.4.1), which predicted the bacterial functional potential [78,79]. Heatmap visualizations were generated using the “pheatmap” package in R to display the predicted functional enzyme profiles [80].

### 2.7. Metabolomic Analysis

#### 2.7.1. Sample Preparation and Extraction

Three individual guts (entire gut) were collected, and six replicates (cages) were performed for each group. The individual gut sample was then extracted for Nuclear Magnetic Resonance (NMR) analysis using a method modified from Gowda and Raftery (2021) [81], Jaikang et al. (2025) [82], and Somtua et al. (2023) [83]. First, cold-distilled water was added to each individual gut and lysis using a tissue homogenizer (LLC Homogenizer, Glas-Col^®^, Terre Haute, IN, USA). The homogenized samples were then filtered using a 0.22 µm filter (Millex^®^-GS Filter Unit, Merck KGaA, Darmstadt, Germany) to remove the pollen residues before metabolite extraction. The metabolites were extracted using a chloroform:water (1:1) solution and then mixed and centrifuged at 2500 rpm for 3 min. The supernatant (upper layer) was collected and processed to remove water using an evaporator and lyophilizer. The dried sample was then re-dissolved in 600 µL of D_2_O containing TSP (Sigma-Aldrich, St. Louis, MO, USA), followed by centrifugation at 15,000 rpm for 5 min, and the supernatant was collected. The supernatant was used to measure metabolites with ^1^H-NMR (Proton nuclear magnetic resonance) Spectroscopy.

#### 2.7.2. ^1^H-NMR Spectroscopy, Peak Analysis, Data Analysis and Metabolite Identification

Metabolite profiling was conducted by the Advanced Scientific Instruments Unit, Faculty of Science, Chiang Mai University, and the Metabolomic Research Group for Forensic Medicine and Toxicology, Department of Forensic Medicine, Faculty of Medicine, Chiang Mai University, Thailand. The analysis was performed using a Bruker AVANCE 500 MHz spectrometer (Bruker, Bremen, Germany) equipped with a Carr–Purcell–Meiboom–Gill (CPMG) pulse sequence (RD—90°, (τ—180°) n—acquire) and presaturation for water suppression. Spectra were acquired at 27 °C, and TSP was used as the internal standard for chemical shift referencing. Further specific technical details, including acquisition parameters, data processing (TopSpin version 4.0.7 and MestReNova version 12.0.0), and metabolite identification methods, followed those described in Jaikang et al. (2025) [82]. In brief, TopSpin software version 4.0.7 was used to identify peaks in the NMR spectra by comparing them to metabolites listed in the Human Metabolome Database (HMDB). Subsequently, MestReNova software version 12.0.0 (MestreLab Research, Santiago de Compostela, Spain) was used to quantify the metabolite relative concentrations, which were reported in micromolar (µM) units. The formula used for metabolite concentration calculations is provided in Jaikang et al. (2025) [82].

#### 2.7.3. Statistical Analysis and Metabolomic Data Analysis

For statistical analysis, the normality of the data was assessed using the Kolmogorov–Smirnov (normality among groups) and Shapiro–Wilk tests (normality between groups). The Kruskal–Wallis test was used to test differences among groups, and the Mann–Whitney *U* test was applied for pairwise comparisons between two groups. *p* < 0.05 was considered statistically significant.

Metabolomic data were analyzed using MetaboAnalyst version 6.0. Prior to analysis, the data were verified, where missing and zero values were removed, followed by filtering out metabolites with a mean intensity below 100. Data normalization was performed using the quantile and median, followed by log_10_ transformation and auto-scaling. Partial Least Squares Discriminant Analysis (PLS-DA) was conducted to differentiate between groups. Model performance was validated using 5-fold cross-validation, goodness-of-fit (R^2^), and predictive ability (Q^2^). Then, to comprehensively assess the metabolic differences between groups, both multivariate and univariate approaches were applied including VIP scores and Volcano plots, respectively. Variable Importance in Projection (VIP) scores were performed to identify potential biomarkers, with metabolites having VIP scores > 1.6 considered significant. Volcano plots were used to visualize significantly altered metabolites, with thresholds set at fold change (FC) > 1.5 and *p* < 0.05. The boxplots displaying the significant differences in metabolites among groups were visualized using the R environment (version 4.4.1) with the “ggplot2” package [67]. Pathway analysis was conducted using the global test method, with relative betweenness centrality as the topology measure and *Apis mellifera* (KEGG) as the reference pathway database. The Kyoto Encyclopedia of Genes and Genomes (KEGG) was also used to determine whether differential metabolites are associated with the host or gut microbiota.

## 3. Results

### 3.1. The Survival Rate and Food Consumption of Apis mellifera

**Figure 2:**
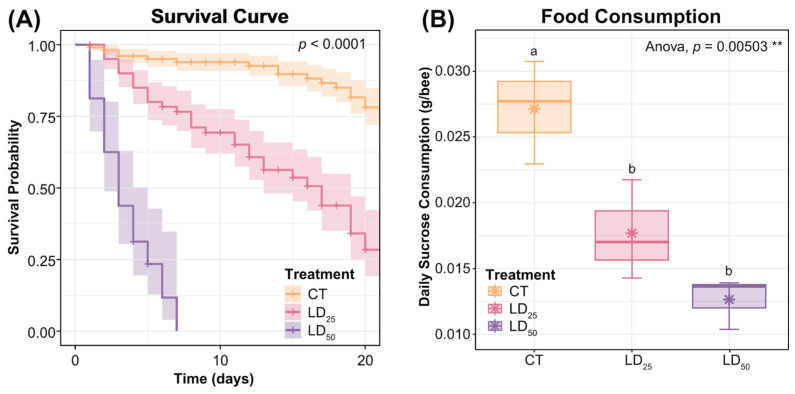
Effects of paraquat exposure on survival and food consumption in *Apis mellifera*. (**A**) Survival curve of *A. mellifera* among groups. Differences between groups were tested using log-rank (*X*^2^) test with Bonferroni correction (*p* < 0.0001). (**B**) Daily sucrose consumption (g/bee) of *A. mellifera* among groups presented as box plots. Values represent sucrose consumption in three cages of surviving bees per group. Differences between groups were tested using one-way ANOVA with Tukey’s correction (*p* = 0.00503). Different alphabets indicate significant differences among groups. Mean value is represented by asterisks. CT represents the control group, LD_25_ represents the sublethal paraquat exposure group, and LD_50_ represents the toxic paraquat exposure group. ** denote a *p*-value of *p* ≤ 0.01.

Oral exposure of *A. mellifera* to paraquat resulted in a high mortality rate. The survival curve showed that the paraquat treatment groups were significantly different from the control group (log-rank test, *X*^2^ = 123, df = 2, *p* < 0.0001) (Figure 2A). Pairwise Bonferroni comparisons also revealed significant differences in survival rates between all treatment groups, with *p* < 0.0001 (Table S1). The control bees can survive up to 40 days (Figure S1). Notably, the toxic dose (LD_50_) caused the highest mortality over time. Similarly, in terms of food consumption, the paraquat treatment groups showed significantly reduced food consumption in *A. mellifera* (Figure 2B). Food consumption differed significantly among treatments (one-way ANOVA, F (2, 6) = 14.51, *p* = 0.00503). Post hoc Tukey’s test showed that both LD_25_ (*p* = 0.0311) and LD_50_ (*p* = 0.0044) consumed significantly less sucrose than the control, whereas LD_50_ did not differ significantly from LD_25_ (*p* = 0.2345).

### 3.2. 16S rRNA Gene Amplicon Sequencing Results

#### 3.2.1. Sequence Read Curation

The raw bacteria amplicon sequence variants (ASVs) from 18 samples resulted in a total of 1,041,319 reads, with a minimum of 46,479 and a maximum of 69,927 reads. After filtering, quality cut-off, denoising, and removing singletons, a total of 608,663 reads remained, with 25,164 and 41,080 as the minimum and maximum reads, respectively. In addition, a total of 376 unique ASVs were identified. Finally, after taxonomic classification and rarefaction analysis, with the removal of mitochondrial and chloroplast sequences and data normalization, the rarefaction curve was generated using the 369 unique ASVs, a total of 452,952 reads, and 25,164 as the minimum and maximum reads. All ASVs are listed, and the rarefaction curves are presented in the Supplementary Material (Table S2, Figure S2).

#### 3.2.2. The Gut Bacteria Community of *Apis mellifera*

The gut bacterial community of *A. mellifera* across the three treatment groups included 4 phyla, 6 classes, 10 orders, 14 families, and 7 genera (Table S3). Relative abundance analysis showed that the honeybee gut microbiota was dominated by the phylum Firmicutes (55%), followed by Proteobacteria (26%) and Actinomycetota (19%) (Table S3).

**Figure 3:**
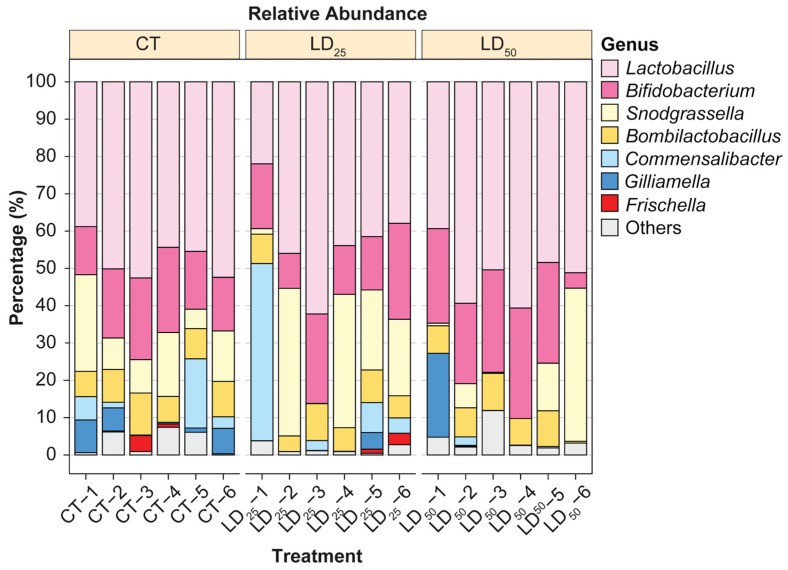
Gut bacterial composition at the genus level. A stacked bar graph shows the relative abundances of gut bacteria, with each bar representing one replicate. Treatment groups include control (CT), sublethal paraquat exposure group (LD_25_), and toxic paraquat exposure group (LD_50_).

At the genus level, *Lactobacillus* was the most abundant taxon, followed by *Bifidobacterium* and *Snodgrassella* (Figure 3). Specifically, the mean relative abundance revealed *Lactobacillus* as the dominant genus (47%), followed by *Bifidobacterium* (19%), *Snodgrassella* (14%), *Bombilactobacillus* (8%), *Commensalibacter* (5%), *Gilliamella* (3%), and *Frischella* (0.6%) (Table S3).

Statistical analysis indicated no significant differences in bacterial composition between the control and paraquat treatment groups, nor between the two paraquat treatment groups (Tables S4 and S5). However, a trend in the relative abundance of *Lactobacillus* was observed between the paraquat treatment groups, with *p* = 0.056 (Table S5).

#### 3.2.3. Diversity of Bacterial Microbiome

**Figure 4:**
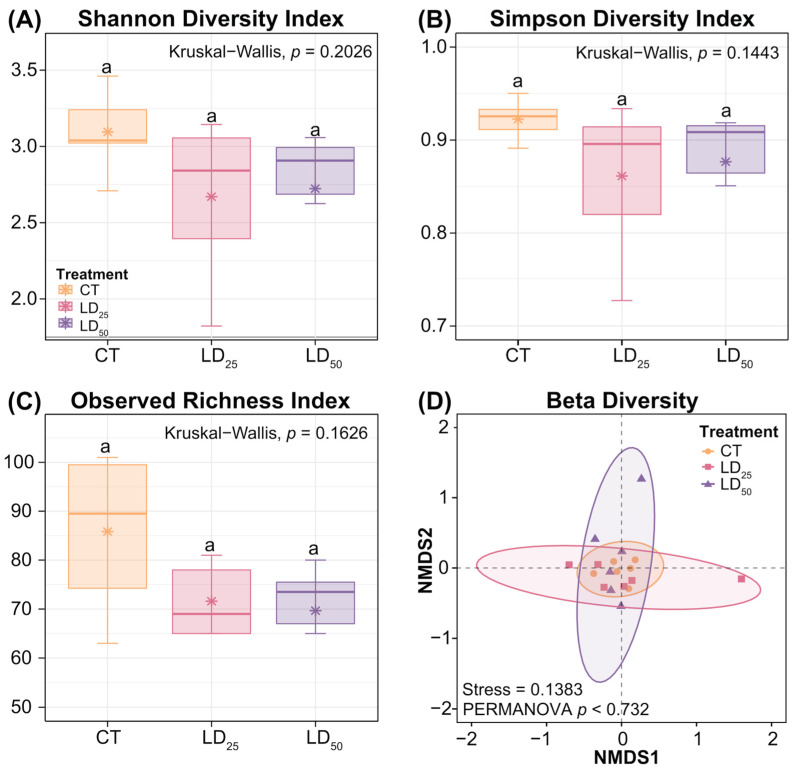
Alpha and beta diversity of gut bacterial communities in *Apis mellifera* following paraquat exposure. Alpha diversity was evaluated using three metrics: (**A**) Shannon, (**B**) Simpson, and (**C**) Observed richness. Differences within treatments are tested using the Kruskal–Wallis test. Different alphabets indicate significant differences among groups. Mean value is represented by asterisks. (**D**) Beta diversity was visualized using NMDS based on Bray–Curtis dissimilarity (stress = 0.1383). Ellipses represent 95% confidence intervals. CT represents the control group, LD_25_ represents the sublethal paraquat exposure group, and LD_50_ represents the toxic paraquat exposure group.

For bacterial microbiome diversity, both alpha and beta diversity showed no significant differences in gut bacterial microbiome in all three treatment groups (Figure 4).

Alpha diversity analysis revealed no significant differences in bacterial communities within the treatment groups (Figure 4A–C). The Kruskal–Wallis test results were *p* = 0.2026 for Shannon (Figure 4A), *p* = 0.1443 for Simpson (Figure 4B), and *p* = 0.1626 for Observed richness (Figure 4C). In addition, the mean and standard deviation (Mean ± SD) for each treatment group across the three alpha diversity metrics supported that the gut bacterial communities were the same (Table S6).

Beta diversity analysis was assessed using non-metric multidimensional scaling (NMDS) based on Bray–Curtis dissimilarity. The NMDS plot (Figure 4D) revealed overlapping clusters among all three groups, suggesting similar bacterial compositions. PERMANOVA showed *p* < 0.732, indicating that the gut bacterial community structure was similar across treatments, with no significant differences in bacterial composition among the groups.

#### 3.2.4. Correlations of Bacterial Microbiome

The correlations of the bacterial microbiome were assessed using network analysis, in which blue edges represent positive correlations, while red edges represent negative correlations. The size of the nodes corresponds to the degree of correlation, with larger nodes indicating stronger correlations and smaller nodes indicating weaker correlations.

**Figure 5:**
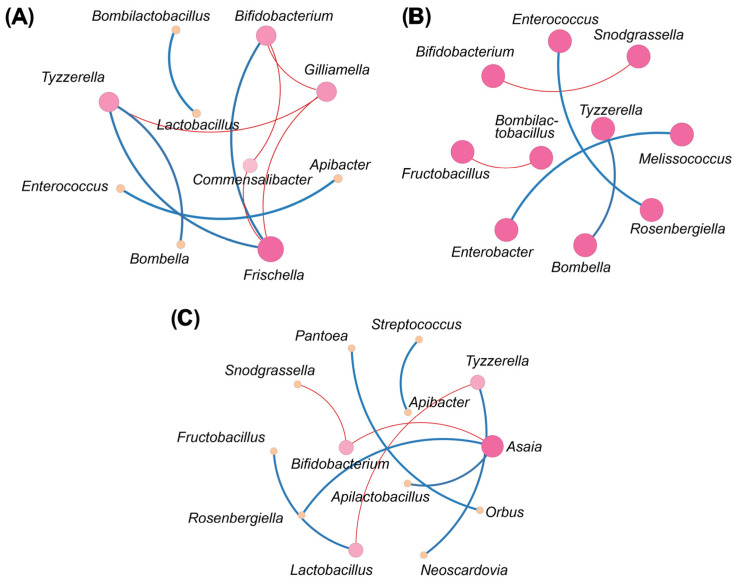
Network analysis of gut bacterial communities in three groups. (**A**) Control group, (**B**) Sublethal paraquat exposure group (LD_25_), (**C**) Toxic paraquat exposure group (LD_50_). The degree of correlations was performed using pairwise correlations at *p* < 0.05 and *r* > 0.7. Blue edges indicate positive correlations, while red edges indicate negative correlations. Each node represents a bacterial genus, where node size and color reflect the correlation strength, ranging from strong (pink) to weak (cream).

For the control group (Figure 5A), network analysis revealed 10 nodes and 10 edges. The network also showed the bacterial communities that reflected a common symbiotic gut microbiota of honeybees. Notably, *Gilliamella* showed the strongest negative correlations with other bacteria, especially with *Frischella*, *Tyzzerella*, and *Bifidobacterium*.

For the sublethal paraquat exposure group (LD_25_) (Figure 5B), network analysis revealed 10 nodes and 5 edges. A positive correlation was observed in *Melissococcus* to *Enterobacter*, whereas negative correlations were shown among symbiosis bacteria, which are *Bifidobacterium*–*Snodgrassella* and *Bombilactobacillus*–*Fructobacillus*.

Similarly, in the toxic paraquat exposure group (LD_50_) (Figure 5C), negative correlations were observed between *Lactobacillus*–*Tyzzerella*, *Bifidobacterium*–*Asaia*, and *Bifidobacterium*–*Snodgrassella*. These symbiotic bacteria were represented by larger nodes, indicating stronger interactions. In addition, the network analysis revealed 13 nodes and 9 edges.

#### 3.2.5. Functional Properties of the Gut Bacteria of *Apis mellifera*

**Figure 6:**
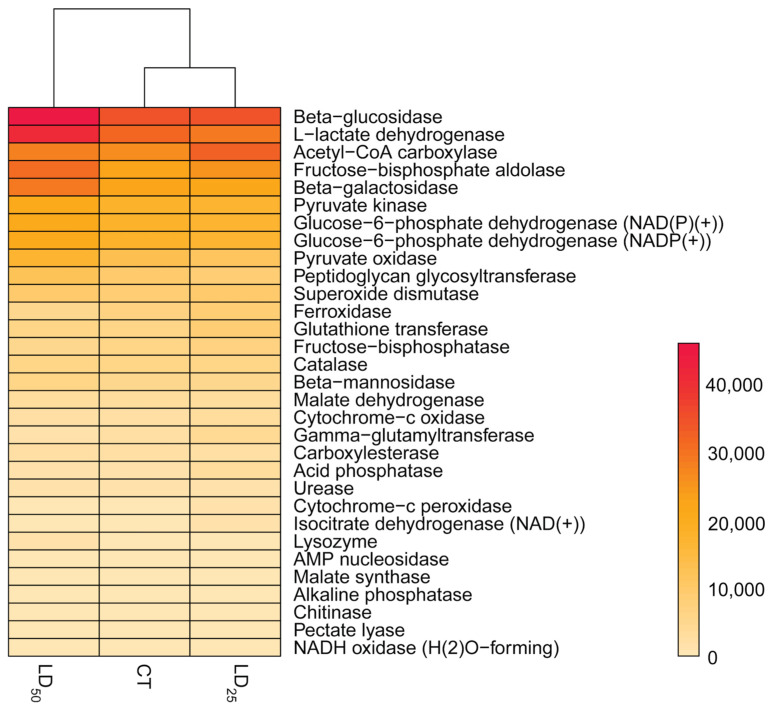
Heatmap visualization of notable enzymes produced by the gut bacteria of *Apis mellifera* under paraquat exposure (LD_25_: sublethal dose; LD_50_: toxic dose) and control (CT) conditions, as predicted using PICRUSt2. Color intensity represents enzyme abundance, ranging from highest (dark red) to lowest (light cream) levels.

Functional prediction analysis revealed that β-glucosidase was a key enzyme in the gut bacterial microbiome of *A. mellifera*, as shown in Figure 6. β-glucosidase showed the highest activity in all three treatment groups, with the highest level observed in the toxic paraquat exposure group (LD_50_). Additionally, notable enzymes involved in amino acid metabolism, energy metabolism, carbohydrate metabolism, and detoxification and oxidative stress defense were shown, for instance, glucose-6-phosphate dehydrogenase, β-galactosidase, L-lactate dehydrogenase, fructose-bisphosphate aldolase, glutathione transferase, catalase, acid phosphatase, lysozyme, malate synthase, alkaline phosphatase, and chitinase.

### 3.3. Metabolomic Results

#### 3.3.1. Overview of Detected Metabolites in *Apis mellifera*

A total of 63 metabolites were detected across all samples and classified into nucleotides, organic acids, amino acids, carbohydrates, lipids, and fatty acids (Table S7). Among these, 11 and 3 metabolites were identified as significantly altered (*p* < 0.05) in the sublethal (LD_25_) and toxic (LD_50_) paraquat exposure groups, respectively, compared with the control group (Tables S8 and S9).

#### 3.3.2. Effects of Paraquat Exposure on the *Apis mellifera* Metabolome: PLS-DA Analysis

**Figure 7:**
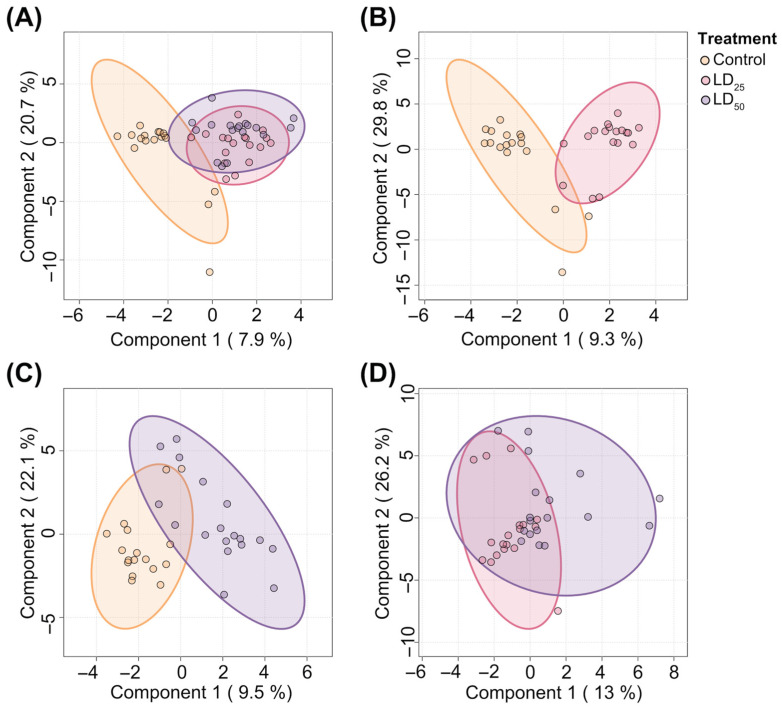
PLS-DA score plots of metabolomic profiles. (**A**) Three-group PLS-DA (CT, LD_25_, LD_50_), where data were normalized by quantile normalization, log_10_ transformation, and auto-scaled. (**B**–**D**) Pairwise PLS-DA comparisons between groups, where data were normalized by median normalization, log_10_ transformation, and auto-scaled. Points represent individual samples, and colors indicate treatment groups: CT (control) in yellow, LD_25_ (sublethal paraquat exposure) in pink, and LD_50_ (toxic paraquat exposure) in purple.

Partial least squares discriminant analysis (PLS-DA) score plots revealed differences in the gut metabolic profiles between the control and paraquat treatment groups. Among the three groups, the PLS-DA score plot, with Component 1 (7.9%) and Component 2 (20.7%), showed separation between the control group and the paraquat treatment groups at both sublethal (LD_25_) and toxic (LD_50_) doses. However, an overlap was observed between the LD_25_ and LD_50_ clusters (Figure 7A). The model performance showed accuracy = 0.54909, R^2^ = 0.79628, and Q^2^ = 0.1638.

To explore these separation trends in more detail, pairwise PLS-DA score plots were performed to assess the potential metabolic variations between groups. For the sublethal paraquat exposure group (LD_25_), the PLS-DA score plot showed separation from the control group, with slight overlap along Component 1 (9.3%) and Component 2 (29.8%) (Figure 7B). The model performance showed accuracy = 0.85833, R^2^ = 0.88506, and Q^2^ = 0.58343. Similarly, the toxic paraquat exposure group (LD_50_) was separated from the control group, although moderate overlap was observed with Component 1 (9.5%) and Component 2 (22.1%) (Figure 7C). The model performance showed accuracy = 0.80476, R^2^ = 0.93721, and Q^2^ = 0.23744. In contrast, the PLS-DA score plot comparing the LD_25_ and LD_50_ groups showed overlapping and clustering together with Component 1 at 13% and Component 2 at 26.2%, with accuracy = 0.49643, R^2^ = 0.38715, and Q^2^ = −0.02835 (Figure 7D).

#### 3.3.3. Significant Metabolites Associated with Paraquat Exposure in *Apis mellifera*

**Figure 8:**
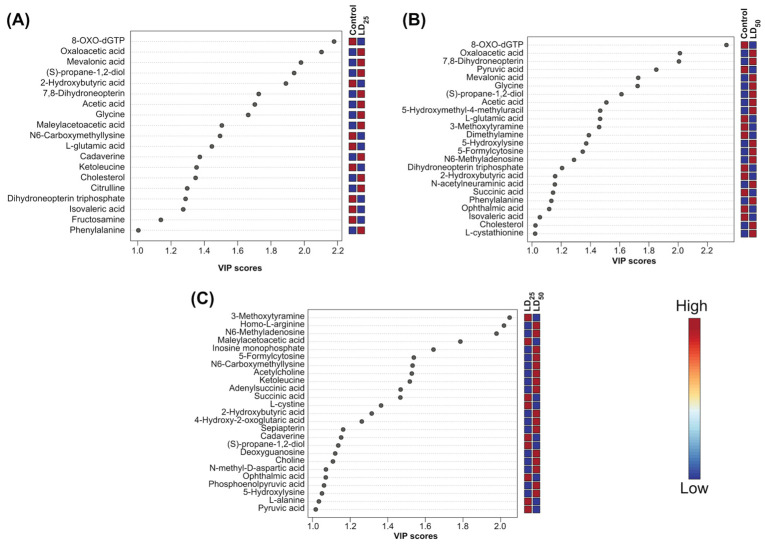
VIP score plots of metabolomic profiles between control and paraquat treatment groups. Data were normalized by median normalization, log_10_ transformation, and auto-scaled (**A**) LD_25_ vs. control. (**B**) LD_50_ vs. control. (**C**) LD_25_ vs. LD_50_. LD_25_ represents sublethal paraquat exposure, while LD_50_ represents toxic paraquat exposure.

To assess potential biomarkers, Variable Importance in Projection (VIP) scores were shown. The VIP score was used to identify the key metabolites that contributed to the class discrimination in each group. The result revealed that eight and seven metabolites showed a VIP score > 1.6 in the LD_25_ and LD_50_ groups, respectively (Figure 8A,B). Both LD_25_ and LD_50_ groups were associated with high levels of oxaloacetic acid (oxaloacetate), mevalonic acid (mevalonate), (S)-propane-1,2-diol (1,2-propanediol), 7,8-dihydroneopterin, acetic acid (acetate), and glycine, along with low levels of 8-oxo-dGTP and 2-hydroxybutyric acid compared with the control. However, pyruvic acid (pyruvate) showed an opposite trend as lower under LD_50_ compared with the control and LD_25_. The VIP score plot comparing the LD_25_ and LD_50_ groups revealed that five metabolites showed a VIP score > 1.6, with 3-methoxytyramine and maleylacetoacetic acid present at higher levels under sublethal exposure (LD_25_), while homo-L-arginine, N6-methyladenosine, and inosine monophosphate were present at higher levels under toxic exposure (LD_50_) (Figure 8C).

**Figure 9:**
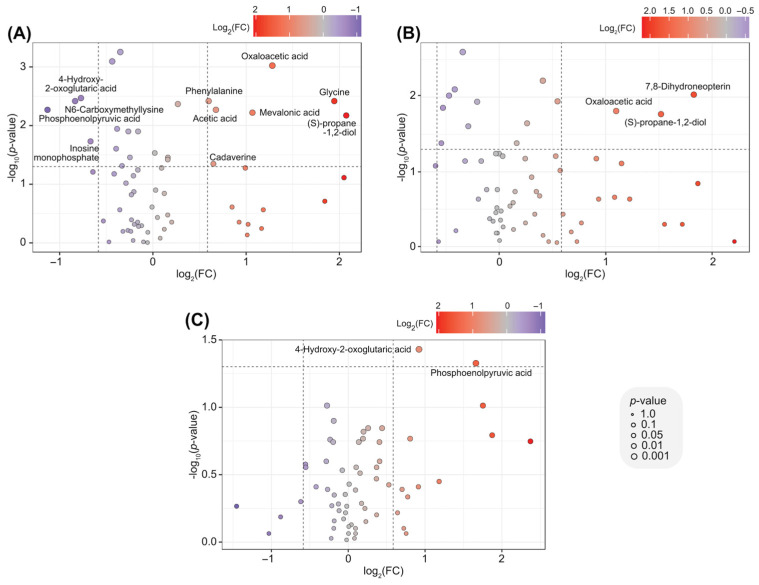
Volcano plots showing significantly differentially regulated metabolites between treatment groups. (**A**) LD_25_ vs. control. (**B**) LD_50_ vs. control. (**C**) LD_25_ vs. LD_50_. LD_25_ represents sublethal paraquat exposure, while LD_50_ represents toxic paraquat exposure. The x-axis represents log_2_ fold change in metabolite abundance, and the y-axis represents −log_10_(*p*-value). Dot size corresponds to the level of significance, and dot color indicates the direction of change (red = upregulated, purple = downregulated).

Although the VIP scores identify the metabolites influential for distinguishing between groups, they do not provide information regarding the direction of metabolic changes. Therefore, to further support the paraquat effect, key metabolites were also classified as upregulated or downregulated based on their fold change and statistical significance in the volcano plot compared with the control group. The volcano plot revealed several metabolites that were significantly altered between groups following paraquat exposure (Figure 9). In the sublethal paraquat exposure group (LD_25_) compared with the control, 11 metabolites were significantly changed, including 7 upregulated and 4 downregulated metabolites (Figure 9A). Among the upregulated metabolites, the expression of glycine and (S)-propane-1,2-diol increased by nearly 2-fold (*p* = 0.0038) and 2-fold (*p* = 0.0067), respectively. Oxaloacetic acid and mevalonic acid increased by 1-fold (*p* = 0.0009 and *p* = 0.0060, respectively), while acetic acid increased by nearly 0.7-fold (*p* = 0.0054), and both cadaverine and phenylalanine increased by 0.6-fold (*p* = 0.0449 and *p* = 0.0038, respectively) (Table S8; Figure 9A).

**Figure 10:**
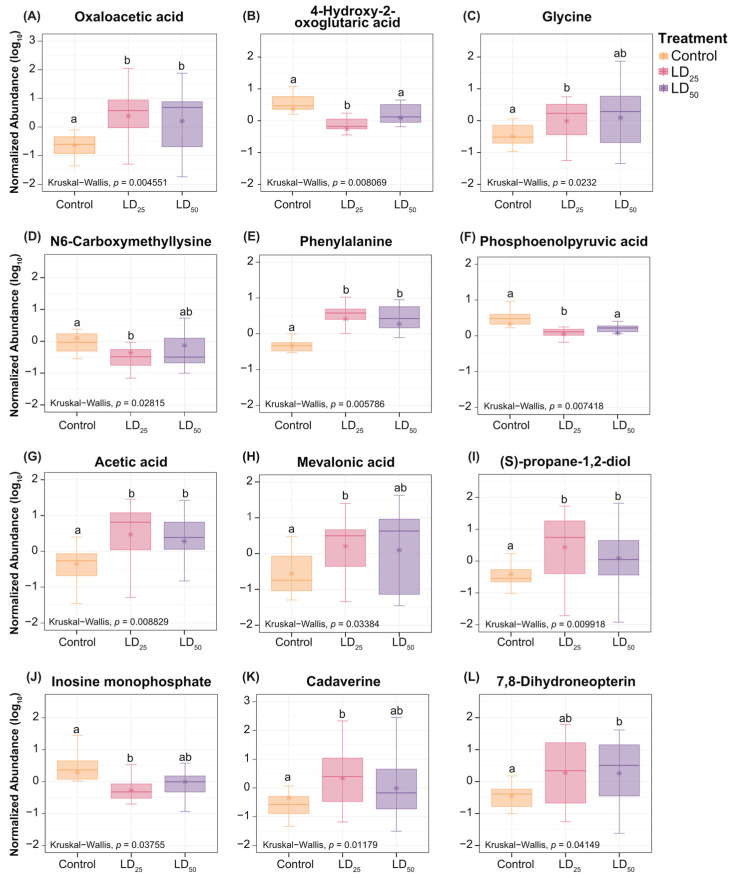
Significant differences in metabolites abundance among groups. (**A**) Oxaloacetic acid (oxaloacetate), (**B**) 4-hydroxy-2-oxoglutaric acid, (**C**) glycine, (**D**) N6-carboxymethyllysine, (**E**) phenylalanine, (**F**) phosphoenolpyruvic acid (phosphoenolpyruvate; PEP), (**G**) acetic acid (acetate), (**H**) mevalonic acid (mevalonate), (**I**) (S)-propane-1,2-diol (1,2-propanediol), (**J**) inosine monophosphate, (**K**) cadaverine, (**L**) 7,8-dihydroneopterin. Group differences were assessed using the Kruskal–Wallis test, with Mann–Whitney *U* tests for pairwise comparisons (*p* < 0.05), as the data did not meet the assumptions of normality required for parametric tests. Comprehensive statistical results, including *p*-values from Mann–Whitney *U* tests for pairwise comparisons, are presented in Table S11. The x-axis represents treatment groups. The y-axis represents normalized metabolite abundance. Different alphabets indicate significant differences among groups. Mean value is represented by asterisks. LD_25_ represents sublethal paraquat exposure group, and LD_50_ represents toxic paraquat exposure group.

In contrast, phosphoenolpyruvic acid (phosphoenolpyruvate; PEP) decreased by 1-fold (*p* = 0.0054), while 4-hydroxy-2-oxoglutaric acid and N6-carboxymethyllysine decreased by nearly 0.8-fold (*p* = 0.0034) and 0.8-fold (*p* = 0.0038), respectively. In addition, inosine monophosphate decreased by 0.7-fold (*p* = 0.0186) (Table S8; Figure 9A). Notably, all 11 metabolites also showed significant differences in abundance (Figure 10A–K).

In the toxic paraquat exposure group (LD_50_) compared with the control, 3 metabolites were significantly upregulated. The expression of 7,8-dihydroneopterin increased by nearly 2-fold (*p* = 0.0093), while (S)-propane-1,2-diol and oxaloacetic acid increased by approximately 1.5-fold (*p* = 0.0169) and 1-fold (*p* = 0.0153), respectively (Table S9; Figure 9B). These metabolites also showed significant differences in abundance (Figure 10A,I,L). In addition, phenylalanine and acetic acid showed significant differences in abundance compared with the control group (Figure 10E,G).

When comparing the LD_25_ and LD_50_ groups, 2 metabolites—4-hydroxy-2-oxoglutaric acid and phosphoenolpyruvic acid were significantly upregulated in the LD_50_ group. Their expression increased by approximately 1-fold (*p* = 0.0371) and nearly 1.7-fold (*p* = 0.0471), respectively, compared with the LD_25_ group (Table S10; Figure 9C). These metabolites also showed significant differences in abundance between the two groups (Figure 10B,F).

#### 3.3.4. Effect of Paraquat on the Metabolomic Pathway of *Apis mellifera*

**Figure 11:**
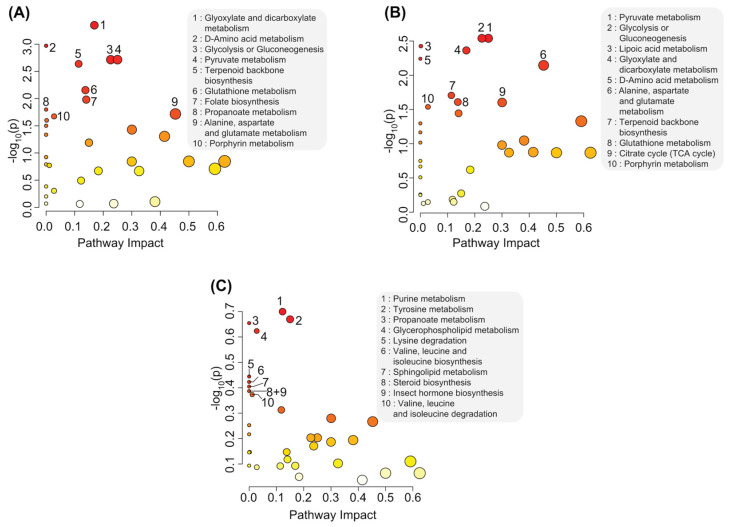
Pathway enrichment analysis. (**A**) LD_25_ vs. control. (**B**) LD_50_ vs. control. (**C**) LD_25_ vs. LD_50_. The x-axis represents the pathway impact, and the y-axis represents the −log_10_ of the *p*-value. Dot color indicates the degree of significance (red = strong significant, light-yellow to white = weak significant).

Paraquat not only altered individual metabolites but also disturbed broader metabolic pathways, as revealed by pathway enrichment analysis. Both sublethal (LD_25_) and toxic (LD_50_) exposures affected 36 pathways, with the top 10 pathways listed in Tables S12 and S13. Among these, eight pathways were shared between the LD_25_ and LD_50_ groups, including “glyoxylate and dicarboxylate metabolism”, “D-amino acid metabolism”, “glycolysis or gluconeogenesis”, “pyruvate metabolism”, “terpenoid backbone biosynthesis”, “glutathione metabolism”, “alanine, aspartate and glutamate metabolism”, and “porphyrin metabolism” (Figure 11A,B). Comparison of the LD_25_ and LD_50_ groups also revealed 36 disturbed pathways, primarily related to carbohydrate, energy, nucleotide, amino acid, and lipid metabolism, as well as insect hormone regulation (Table S14, Figure 11C).

## 4. Discussion

In this study, we examined the effects of sublethal and toxic doses of paraquat on *A. mellifera* using both 16S rRNA gene amplicon sequencing and metabolomic analysis. These approaches provided insights into both upstream (gut microbiome) and downstream (metabolic) biological processes and improved the understanding of honeybee responses to paraquat-induced stress. The experiment was conducted under controlled laboratory conditions to minimize environmental variability. Furthermore, the use of a sucrose-only diet, as standard condition in toxicological assays, reduced dietary influences and other potential confounding factors. Consequently, the observed biological responses and metabolic alterations could be attributed primarily to paraquat exposure.

### 4.1. Paraquat Impact on the Survival and Food Consumption of Apis mellifera

Our findings demonstrated that paraquat exposure significantly reduced the survival and sucrose consumption of *A. mellifera* in a dose-dependent manner, with the toxic dose (LD_50_) causing the highest mortality and decline over time. These results are consistent with previous studies reporting that both *A. mellifera* and *A. cerana* were highly susceptible to paraquat, with higher concentrations of paraquat leading to increased mortality and reduced sucrose intake [16,41,42]. Similar outcomes have also been observed for glyphosate, another widely used herbicide, in which elevated concentrations were associated with mortality, limited recovery, and a reduction in sucrose consumption [45,47,48,84,85]. The reduced sucrose consumption and increased mortality observed in paraquat-exposed *A. mellifera* can be attributed to multiple interacting mechanisms, including altered feeding behavior [33,84,85,86,87,88,89,90], sensory detection of toxins [86,89,91], molecular stress responses [16,40,42,92,93], and cellular damage [92,93,94,95]. Paraquat is known to act as an oxidative stressor that can damage digestive cells in the midgut [92,94,95], as reported in Wang et al. (2024) [95] and Farder-Gomes et al. (2024) [93], where insecticides and herbicides cause structural damage to the midgut; as the honeybee gut is essential for nutrient digestion and absorption, such damage can impair nutrient uptake and induce starvation [43,44,96], ultimately contributing to reduced sucrose consumption and increased mortality. Notably, the control bee group survived for up to 40 days, supporting the adequacy of the baseline conditions and suggesting that malnutrition might be associated with paraquat stress. Therefore, these explain the strong reductions in sucrose consumption observed in our study, as toxin-sensitive receptors in the mouthparts and midgut were likely activated more extensively under paraquat exposure.

### 4.2. Characterization of the Gut Bacterial Community, Diversity and How They Response to Paraquat Exposure

Our results showed no significant differences in gut bacterial diversity or overall community composition between paraquat treatment groups and the control, as supported by both α- and β-diversity analyses. The gut microbiome of *A. mellifera* was dominated by typical core symbiotic bacteria, including *Lactobacillus*, *Bombilactobacillus*, *Snodgrassella*, *Bifidobacterium*, and *Gilliamella*, with lower abundances of non-core taxa such as *Frischella* and *Commensalibacter* [43,44,60,96,97,98]. These core taxa collectively account for approximately 95% of the honeybee gut microbiome and play essential roles in host nutrition, immunity, pathogen defense, and overall colony health [43,44,60,96,97,98]. Because the gut microbiome is critical for honeybee health, its disruption by xenobiotics can lead to negative effects on the honeybee host. However, our findings did not reveal significant alterations in gut microbiome composition following paraquat exposure, contrasting with previous studies that reported microbiome disturbances in honeybees exposed to herbicides, particularly glyphosate. For example, previous studies have reported significant reductions in core symbiotic bacteria such as *S. alvi*, *Lactobacillus*, and *Bifidobacterium* within 3–7 days following glyphosate exposure in *A. mellifera* and bumblebee species [45,46,47,48,51]. Some alterations in relative abundance were observed in our study, including reduced *S. alvi* at LD_50_ compared with LD_25_, lower *Bombilactobacillus* abundance in both paraquat treatment groups, and an increase in *Lactobacillus* at LD_50_ with a near-significant difference between paraquat treatment groups. However, these changes were not statistically significant and did not lead to detectable alterations in overall community composition.

The absence of significant paraquat-induced changes may be explained by several factors, including the exposure duration [46,47,48,50,99], xenobiotic type or concentration [46,47,48,50,99,100,101], species-specific sensitivity [42,46,47,51,52], and the toxicological mechanism of paraquat [42,92,94]. Previous studies indicate that microbiome alterations often emerge after prolonged or repeated glyphosate herbicide exposure. Differences in exposure duration may account for the discrepancy between our findings and those reported in previous studies. In the present study, paraquat exposure was assessed at LD_25_ and LD_50_ doses, referencing the kinetics reported by Phokasem et al. (2024) [42], which represent the acute exposure at which paraquat toxicity started to occur. This acute exposure likely induces rapid oxidative stress, tissue damage, and physiological collapse [42,92,94,95], leading to mortality before the microbiome restructuring occurs. Consequently, while paraquat primarily affects host physiology in the acute phase, microbiome shifts might only emerge under more chronic exposure scenarios [46,47,50]. Therefore, longer exposure durations with increased experimental replication may be required to detect paraquat-induced changes in the microbiome. In addition, both bee species and gut bacterial taxa can exhibit variable responses to xenobiotics, reflecting differences in host sensitivity [42,52], and bacterial resistance mechanisms [46,47,51]. Collectively, these findings suggest that paraquat may primarily affect honeybee host physiology, with secondary or delayed effects on the gut microbiome. Moreover, the shifts may emerge under prolonged exposure, warranting further investigation.

### 4.3. Altered Correlations in the Gut Microbiome Following Paraquat Exposure

The honeybee gut microbiome forms a complex ecological network in which interactions among symbiotic bacteria collectively influence host physiology and overall health. Network analysis revealed that the control group exhibited a balanced bacterial network, characterized by comparable proportions of positive and negative correlations. This pattern indicates a stable gut ecosystem that supports honeybee health. Overall, bacteria in the network were primarily involved in core symbiotic bacteria, particularly lactic acid bacteria, along with some non-core members. Additionally, core symbionts typically showed positive associations with other bacteria, while these bacteria play a significant role in food digestion, nutrient acquisition, appetite regulation, pathogen defense, stress resistance, and boost the immune system [97,102,103]. *Gilliamella*, a core bacterium, exhibited the strongest negative correlations with other taxa. *Gilliamella* has been reported to exhibit antagonistic activity toward other microbes, which might associate with the honeybee’s immunity and contribute to host health [60,97,103]. Therefore, the overall microbial network in the control group demonstrated well-balanced connectivity, indicative of a stable gut environment that supports normal honeybee health.

In contrast, the microbial network structure of the paraquat-exposed groups revealed an imbalance of positive and negative correlations. This altered pattern is consistent with Zhang et al. (2025) [104], who reported that the insecticide, ethiprole, disrupted gut microbial networks in *A. mellifera* L., suggesting that xenobiotic stressors can alter the usual correlation patterns in the honeybee microbiome. Our results indicate that core bacteria exhibit altered correlations under paraquat exposure. Specifically, negative correlations were observed among core symbiont pairs, including *Bifidobacterium*–*Snodgrassella* and *Bombilactobacillus*–*Fructobacillus*, as well as between core and non-core taxa, including *Lactobacillus*–*Tyzzerella* and *Bifidobacterium*–*Asaia*, compared with predominantly positive associations in the control network. These core taxa are typically associated with immunity, detoxification, xenobiotic resistance, and pathogen defense [97,102,103,104], and their shift toward negative associations suggests the disruption of cooperative correlations within the gut microbiome. At the same time they were associated with a higher proportion of non-core symbionts, environmental taxa, and low-abundance bacteria compare with the control group. Notably, pathogenic taxa such as *Enterobacter*, *Pantoea,* and *Melissococcus* appeared in the network and exhibited positive correlations. Both *Enterobacter* and *Pantoea* have been reported to cause changes in network connectivity and overall gut stability, and *Enterobacter* can be a pathogen in honeybees, leading to increased mortality [43,105]. *Melissococcus plutonius*, a bacterium that causes European foulbrood (EFB), has been detected in adult honeybees [106]. Additionally, *Enterococcus* and *Apilactobacillus* showed positive correlations within the paraquat-exposed networks, and these taxa have been reported to mitigate the negative effects of insecticides in honeybees [102,107]. Their correlation may indicate a microbial response aimed at counteracting paraquat-induced stress.

Collectively, these findings suggest that paraquat exposure may be associated with perturbations in altered microbial network structure, characterized by the increased functional activity of potentially pathogenic or opportunistic bacteria and negative correlations among beneficial symbionts. These network changes may be linked to reduced food consumption following paraquat exposure, as a nutritional deficiency, which might influence changes in correlations among gut bacteria. Furthermore, although paraquat exposure appeared to alter microbial correlation networks, it did not significantly affect overall bacterial diversity or community composition. This suggests that paraquat may influence microbial interactions before detectable changes in community structure occur.

### 4.4. Functional Role of Gut Bacterial Enzymes in Apis mellifera During Paraquat Exposure

Gut bacteria play a significant role in promoting honeybee behavior, survival, and health, normally through the activities of bacterial enzymes. In this study, we assessed the functional role of the gut microbiome and found that the control group and the sublethal paraquat group (LD_25_) clustered within the same clade, whereas the toxic exposure group (LD_50_) formed a distinct clade. This finding suggests that the LD_50_ group showed a stronger effect on honeybees and that acute exposure may induce enhanced or long-lasting microbial enzymatic potential, as reflected in their distinct expression patterns.

Most of the enzymes identified in this study were associated with host health, including digestion, immune defense, detoxification, and energy metabolism [60,108,109,110,111,112]. Notably, β-glucosidase, β-galactosidase, L-lactate dehydrogenase, and fructose-bisphosphate aldolase were more abundant in the toxic exposure group (LD_50_). β-glucosidase, an enzyme involved in immune defense [60], showed a high level, suggesting its role in enhancing the host’s resistance to paraquat stress. Similarly, β-galactosidase, which contributes to nutrient acquisition by digesting polysaccharides and pectin, was also increased. Its high level under toxic exposure (LD_50_) may reflect reduced sucrose intake, where honeybees respond by attempting nutrient digestion to support survival. In addition, L-lactate dehydrogenase, an enzyme associated with energy production and metabolic regulation, was also increased. This finding is consistent with a previous study reporting that paraquat induces *A. mellifera* to increase the level of lactate dehydrogenase [109]. Therefore, the paraquat impact on lactate dehydrogenase level is consistent with a stress-response mechanism and enhances the energy of the honeybee. Finally, fructose–bisphosphate aldolase, an enzyme in the glycolysis that supports energy metabolism [108,111], also exhibited a high level in the toxic exposure group (LD_50_), suggesting that honeybees enhance glycolysis activity to sustain energy production and try to survive under paraquat stress.

### 4.5. Metabolic Alterations in Apis mellifera Following Paraquat Exposure

Paraquat exposure is well known to induce oxidative stress through the excessive production of reactive oxygen species (ROS), leading to mitochondrial dysfunction, cellular damage, and neuroinflammatory processes that contribute to accelerated aging and increased mortality [92,94]. Both sublethal (LD_25_) and toxic (LD_50_) paraquat exposures altered key metabolic pathways, suggesting the activation of common stress-response and detoxification mechanisms, indicating that paraquat exposure triggered systemic metabolic reprogramming in *A. mellifera*. Although the LD_25_ and LD_50_ groups partially overlapped in the PLS-DA model, the observed metabolic responses across treatment groups highlight the dose-dependent effects of paraquat on honeybee metabolism. Nevertheless, some limitations should be noted. In some PLS-DA models, lower R^2^ and Q^2^ values were observed, due to the inherent biological variability in samples and limited replication. Therefore, increasing the biological replication in future study would enhance the model’s robustness. Moreover, individual variability in honeybee stress tolerance is uncontrolled in living systems and may influence metabolic responses.

#### 4.5.1. Central Carbon and Energy Metabolism

Paraquat exposure significantly affected pathways involved in central carbon metabolism, particularly glycolysis, gluconeogenesis, and the citrate cycle (TCA). These metabolic pathways play central roles in energy production, cellular respiration, and the generation of metabolic precursors and are associated with immune regulation, stress responses, and antioxidant defense mechanisms [113,114,115,116,117]. Consistent with this, several key intermediates, including oxaloacetic acid (oxaloacetate), pyruvic acid (pyruvate), and phosphoenolpyruvic acid (phosphoenolpyruvate; PEP), were altered following paraquat exposure. Additionally, (S)-propane-1,2-diol, acetic acid (acetate), glycine, and phenylalanine were also affected.

Several metabolic alterations were observed within the central carbon metabolism. Pyruvic acid represents a central metabolic hub linking glycolysis, gluconeogenesis, and the TCA cycle. In the present study, honeybees exposed to a sublethal (LD_25_) dose of paraquat exhibited higher pyruvic acid levels than those exposed to a toxic (LD_50_) dose. In contrast, pyruvic acid levels were reduced in LD_50_ group compared with the control group. Pyruvic acid is known to play a key role in energy metabolism, being a fuel source for mitochondria, and detoxification by enhancing energy availability to promote insect resistance to xenobiotic stress [114,115,118]. Therefore, the high abundance of pyruvic acid suggests enhanced energy metabolism under oxidative stress, in which the sublethal dose (LD_25_) appears to induce adaptive metabolic responses by activating energy and detoxification pathways aimed at survival. In contrast, the lower abundance of pyruvic acid observed in the LD_50_ group may indicate a distinct metabolic response under severe paraquat toxicity, although the underlying mechanisms remain unclear.

Furthermore, oxaloacetic acid, another key intermediate in central carbon metabolism, was also altered following paraquat exposure. In our results, both paraquat doses showed the upregulation of oxaloacetic acid, and this metabolite plays a key role in maintaining TCA cycle flux [115,116,119]. Similar metabolic responses have been reported in honeybees exposed to other xenobiotics. Consistent with our findings, *Apis mellifera* exposed to insecticide for three days showed significant upregulation of oxaloacetic acid, along with metabolites and enzymes involved with glycolysis, the TCA cycle, and oxidative phosphorylation [116]. Notably, the increased abundance of glycine and phenylalanine observed in honeybees exposed to a sublethal (LD_25_) dose may further support a metabolic response, as the catabolism of these amino acids contributes substrates to pyruvic acid and TCA cycle-related pathways [118]. Elevation of glycine and phenylalanine has also been reported in honeybees exposed to insecticides or herbicides, and these changes have been associated with nervous system responses to herbicide exposure [120,121,122,123]. Collectively, the altered abundance of oxaloacetic acid, glycine, and phenylalanine indicates perturbations in central carbon and amino acid metabolism in response to paraquat exposure.

Paraquat-induced alterations in the central carbon metabolism are also linked to glycolysis and gluconeogenesis, as PEP can be converted to pyruvic acid in glycolysis and oxaloacetic acid associated with the TCA cycle and gluconeogenesis through its conversion to PEP by phosphoenolpyruvate carboxykinase (PEPCK) [115,124,125]. In this study, PEP expression was observed to alter under paraquat exposure. Phosphoenolpyruvic acid is a key metabolite involved in glycolysis, glucose regeneration (gluconeogenesis) and energy production [115,124,125], which are essential for honeybees due to their known high carbohydrate and energy demands for activities [126]. Notably, PEP is associated with the pentose phosphate pathway (PPP), although PEP is not a direct intermediate of the PPP. Its production through gluconeogenesis can contribute to glucose regeneration, which subsequently provides precursors, as the entry substrate for the PPP [125]. This linkage may support NADPH production and antioxidant defense under paraquat-induced oxidative stress. Previous studies have shown that stress and oxidative stress can stimulate PEPCK activity and promote PEP production in rat, roundworm, and bug [124,125,127], and increased PEP can reduce susceptibility and enhance the survival capacity under stress [124,125]. These findings suggest that oxidative stress can activate PEPCK and increase PEP synthesis as part of a metabolic response. In contrast to these reports, our results showed that PEP was downregulated under the sublethal paraquat dose (LD_25_), whereas no significant change was observed at the toxic dose (LD_50_) compared with the control. However, the PEP levels at LD_50_ were higher than those observed at LD_25_. These findings suggest a dose-dependent metabolic shift in response to paraquat-induced oxidative stress. Under sublethal exposure (LD_25_), the downregulation of PEP was consistent with the high abundance of pyruvic acid, suggesting a metabolic shift toward enhanced glycolytic flux and energy production. At the same time, elevated ROS may disrupt PEP-associated metabolic processes, limiting gluconeogenic capacity and reducing cellular protection against oxidative stress. These findings indicate that paraquat exposure not only leads to stimulating energy production in the host to support stress responses but also impairs metabolic regulation. In contrast, although gluconeogenic activity may be partially maintained at LD_50_, this compensatory response may be insufficient, due to the high sensitivity of honeybees and the substantial physiological impairment caused by paraquat toxicity, leading to honeybee mortality before function.

Furthermore, paraquat also disturbed the glyoxylate and dicarboxylate metabolism. Paraquat altered 4-hydroxy-2-oxoglutaric acid, an intermediate in glyoxylate and dicarboxylate metabolism, which can be converted to glyoxylate. Glyoxylate subsequently feeds into the production of metabolites-associated glycolysis and the TCA cycle intermediates such as oxaloacetic acid [119,128]. In our results, 4-hydroxy-2-oxoglutaric acid exhibited a dose-dependent response to paraquat exposure. Honeybees exposed to the sublethal dose (LD_25_) showed downregulation of this metabolite compared with the control, whereas the toxic dose (LD_50_) showed no significant difference from the control but remained higher than LD_25_. Similar alterations have been reported in Mrinalini et al. (2015) [129]; they reported that *Nasonia vitripennis* venom altered 4-hydroxy-2-oxoglutaric acid levels in *Sarcophaga bullata* pupae. Together, these observations suggest that foreign stressors, such as pathogens, toxins, and xenobiotics, can disrupt this metabolite and alter host metabolic processes and responses. 

#### 4.5.2. Nucleotide and Stress-Associated Metabolism

Paraquat exposure also disrupted nucleotide and purine metabolisms, further indicating altered energy balance and cellular stress responses. Nucleotide and purine metabolisms are essential for cellular energy transfer, DNA/RNA synthesis, DNA repair, and antioxidant activity, supporting cellular energy production and contributing broadly to host physiological function [117,130]. Our results revealed that after honeybees were exposed to a sublethal dose of paraquat (LD_25_), the level of inosine monophosphate (IMP) was downregulated, while IMP is a key precursor of ATP- and GTP-related nucleotides [117,130]. However, the synthesis of purine nucleotides is highly energy-demanding [117]; so, the reduction in IMP may reflect paraquat-induced stress, leading to decreased sucrose intake and insufficient energy availability. Consequently, reduced IMP levels may constrain nucleotide biosynthesis, potentially impairing DNA repair, cell proliferation, and overall recovery capacity.

Interestingly, both paraquat treatments showed reduced levels of 8-oxo-dGTP, an oxidized nucleotide formed under oxidative stress that can induce mutagenesis when incorporated into DNA, ultimately impairing cellular physiology and brain function [131,132]. Despite paraquat typically increasing oxidative damage, the lower accumulation of 8-oxo-dGTP suggests the activation of cellular defense mechanisms that remove oxidized nucleotides before DNA incorporation. In addition, reduced nucleotide precursor availability, as indicated by lower IMP levels, may further limit substrate availability for oxidation. Together, these findings indicate that paraquat exposure affects purine and nucleotide metabolism, although the mechanisms underlying the reduced accumulation of 8-oxo-dGTP require further investigation.

Furthermore, both paraquat treatments were associated with increased levels of 7,8-dihydroneopterin, with significant upregulation observed under the toxic dose (LD_50_). However, the function of 7,8-dihydroneopterin has not yet been reported in honeybees. Previous studies have identified 7,8-dihydroneopterin in the roundworm (*Caenorhabditis elegans*), where decreased levels were observed following exposure to the herbicide atrazine, suggesting a potential association with reproductive impairment. In humans, 7,8-dihydroneopterin was a biomarker of immune activation, inflammation, oxidative stress, and cellular damage in biological systems [133]. The accumulation of 7,8-dihydroneopterin observed in this study may indicate a response to stress associated with paraquat-induced oxidative damage. Additionally, 7,8-dihydroneopterin functions as an antioxidant, accumulates at sites of inflammation and tissue damage, and associates with macrophages [133]. This response associated with macrophages may also be linked to stress-related signaling pathways, such as the Jun N-terminal kinase (JNK) pathway, which is known to regulate tissue recovery and stress responses in *Drosophila* DNA damage [134].

#### 4.5.3. Nutritional and Hormone-Related Metabolism

Metabolites associated with glucose sensitivity and nutritional status were also altered following paraquat exposure. Our results showed a low accumulation of 2-hydroxybutyric acid in both paraquat doses, with N6-carboxymethyllysine significantly downregulated in the sublethal dose (LD_25_) compared with the control. However, the presence and roles of these metabolites in honeybees remain unclear, as no studies have reported their occurrence in this species. Notably, research on *Drosophila* has identified N6-carboxymethyllysine as a metabolite with increased levels following feeding, suggesting a connection to postprandial metabolic processes [135]. In humans, these two metabolites are recognized biomarkers of insulin resistance and type 2 diabetes, where elevated levels are typically associated with chronically high blood glucose [136,137]. The paraquat-exposed honeybees consumed less sucrose, suggesting reduced sugar uptake and lower intracellular glucose in their cells. Although their functional and physiological significance in honeybees remains unclear, the reduced abundance observed in this study was consistent with the lower sucrose consumption recorded in paraquat-exposed honeybees. Together, these findings suggest that paraquat exposure may influence metabolic processes associated with nutrient utilization and energy metabolism.

Beyond metabolic and nutritional reprogramming, paraquat exposure may also disrupt higher-level physiological regulation, particularly hormone-mediated pathways. Paraquat exposure also appeared to disrupt insect hormonal regulation, particularly pathways associated with terpenoid backbone biosynthesis. In our study, mevalonic acid (mevalonate), a key intermediate of the mevalonate pathway [138], was elevated under both paraquat doses, with significant upregulation at the sublethal dose (LD_25_). The mevalonic acid and mevalonate pathway is directly linked to the biosynthesis of juvenile hormone III (JH3), an essential gonadotrophic hormone in honeybees that regulates various physiological processes, including growth, development, reproduction, and life cycle [95,139,140,141]. Juvenile hormone III is also associated with honeybee social interaction and colony communication within the hive environment [142], where the reduced sucrose consumption and increased mortality observed under paraquat exposure, with the absence of a hive social environment, may also reflect the stress-associated disruption of social interaction, potentially contributing to activation of the mevalonate pathway and JH3. Moreover, JH3 has also been associated with antioxidant defense and increased tolerance to insecticides [95,143,144,145]. Therefore, the observed increase in mevalonic acid may reflect the activation of a protective hormonal response aimed at enhancing JH3 synthesis under paraquat-induced oxidative stress. Consistent with this interpretation, Wang et al. (2024) [95], demonstrated that JH3 supplementation can mitigate insecticide-induced midgut damage and improve stress tolerance in *A. cerana*. However, contrasting responses of the mevalonate pathway have been reported across species and stressors [95,146]. In the same study, JH3 abundance decreased in *A. mellifera* following insecticide exposure [95], while Erban et al. (2019) [146] reported suppression of the mevalonate pathway in *Bombus terrestris*. These findings suggest that hormonal regulation via the mevalonate pathway is stressor and species-specific. Collectively, these results indicate that the increase in mevalonic acid may be associated with tissue repair processes, particularly in the midgut, which is highly susceptible to oxidative damage induced by paraquat [92,94,95]. In addition, this response may reflect the activation of hormone-mediated protective mechanisms that support physiological function under stress conditions.

#### 4.5.4. Host–Microbiome Metabolic Interactions

Our findings also highlight metabolic responses from the interaction between the honeybee host and its gut microbiome. Honeybees primarily obtain amino acids from pollen [118,147], while the gut microbiome can also contribute to amino acid biosynthesis through pollen digestion [148]. This is particularly important under conditions of nutritional deficiency, where enhanced amino acid metabolism supports nitrogen homeostasis and promotes survival under reduced dietary consumption [149], which is consistent with our results. Notably, the increased abundance of amino acids, SCFAs, and pyruvic acid observed in this study may reflect metabolic contributions from gut symbionts and their associated enzymes [150,151,152]. The gut microbiome can contribute to amino acid biosynthesis through the enzymatic degradation of pollen components [148]. The increased levels of glycine and phenylalanine observed in the paraquat-exposed group may be explained by the microbial carbohydrate-degrading enzymes, including β-glucosidase and β-galactosidase, which are known to facilitate pollen breakdown [153], consistent with our result. Furthermore, the gut symbiont bacterium *Gilliamella* has been reported to contribute to host stress adaptation [151], and *G. apicola* can produce pyruvic acid and other metabolites that support host energy metabolism, stress tolerance, and survival [150]. Gut bacteria also encode L-lactate dehydrogenase, which interconverts pyruvic acid and lactate linked to carbohydrate metabolism and glycolysis, supporting microbial and host energy production [154,155]. The results might demonstrate a correlation between the microbial L-lactate dehydrogenase potential and the observed elevation of pyruvic acid levels in the metabolome. The consistent alignment between functional predictions and metabolomic data support the gut microbiome’s role in modulating host energy homeostasis under paraquat stress. In addition, some metabolites identified in this study, including (S)-1,2-propanediol and acetic acid (acetate), a short-chain fatty acid (SCFA), were commonly produced by gut bacteria such as *Lactobacillus* [152,156,157]. These metabolites can enter host metabolic pathways and contribute to energy production and host physiology [152].

Consistent with the microbial contribution to host metabolism, sublethal paraquat exposure (LD_25_) in our study showed the upregulation of acetic acid. Acetic acid is produced through sugar fermentation and carbohydrate metabolism by acetic acid bacteria (AAB) and lactic acid bacteria (LAB) [152,156,157,158,159], including genera such as *Lactobacillus*, *Bifidobacterium*, *Commensalibacter*, *Bombella*, and *Asaia*, which are known to colonize in the honeybee gut and are adapted to acidic and nutrient-limited environments [158,159,160]. Previous studies have reported that acetic acid accumulates at the highest levels in the ileum of adult honeybees [152,160] and can also exhibit antifungal properties [160]. Moreover, acetic acid can be converted into acetyl-CoA and enter the TCA cycle [161]. Therefore, the elevated acetic acid observed under sublethal paraquat exposure may reflect altered host–microbiome metabolic interactions under nutritional stress from paraquat, which leads to linking microbial metabolites to host energy metabolism under oxidative stress conditions.

Propanoate metabolism also appeared to play an important role in the paraquat response. The increased levels of (S)-1,2-propanediol (1,2-propanediol), a precursor of propanoate [162,163] at both paraquat doses, suggest enhanced microbial fermentation and host metabolic adaptation under stress conditions. Notably, (S)-1,2-propanediol can be produced by gut bacteria such as *L. buchneri* and related species during carbohydrate fermentation under anaerobic conditions, as in the honeybee gut [156,157]. Additionally, propanoate, an SCF, can be produced by core symbiotic bacteria in the honeybee gut, such as *G. apicola* and *Lactobacillus*, with high accumulation of propanoate reported in the rectum of *A. mellifera*, and it can be synthesized through multiple metabolic routes [152]. Insects, including *A. mellifera*, have a metabolic pathway in which propanoate is catabolized into 3-hydroxypropionate and subsequently converted to acetic acid [164]. This acetic acid can be further transformed into acetyl-CoA. The intermediate subsequently feeds into the TCA cycle and contributes to cellular energy production [161]. Therefore, the upregulation of (S)-1,2-propanediol after exposure to paraquat, at both sublethal (LD_25_) and toxic (LD_50_) doses, as well as the response of propanoate metabolism in this study, may reflect enhanced microbial fermentation and the activation of propanoate metabolism, potentially providing additional metabolic substrates that support host energy production under paraquat-induced stress.

Collectively, paraquat-induced oxidative stress leads to severe physiological disruption in honeybees, including tissue damage, metabolic and energy dysfunction, and increased mortality. Evidence of tissue degradation was supported by the upregulation of cadaverine, a metabolite associated with tissue decomposition and microbial activity [165,166,167,168]. Cadaverine is typically produced through lysine decarboxylation during protein hydrolysis by bacteria such as *Pseudomonas*, *Enterobacter*, *Enterococcus*, and *Bacillus* [165,166,167], some of which were increased under paraquat exposure. Notably, in our study, sublethal paraquat exposure (LD_25_) resulted in the upregulation of cadaverine, along with the bacteria *Enterobacter* and *Enterococcus*. This co-occurrence suggests that paraquat-induced tissue damage may promote microbial proliferation and metabolic activity, contributing to cadaverine accumulation. These findings highlight a coordinated host–microbiome metabolic response that jointly contributes to tissue degradation under stress conditions.

Together, the findings suggest that paraquat exposure reduces survival and food consumption in *A. mellifera*. Furthermore, although the composition of the gut microbiome remains unchanged, paraquat exposure might alter the microbial network structure and induce dose-dependent metabolic alterations, affecting pathways related to central carbon metabolism, amino acid metabolism, nucleotide biosynthesis, and terpenoid biosynthesis. Notably, some metabolic changes were more pronounced in honeybees exposed to the sublethal dose (LD_25_) than in those exposed to the toxic dose (LD_50_), indicating that metabolic responses differed according to exposure intensity. This suggests that the sublethal dose may have triggered a metabolic response to paraquat-induced stress through alterations in metabolites associated with energy metabolism and oxidative stress, thereby supporting survival. In contrast, the toxic dose might experience higher oxidative stress, potentially limiting or altering metabolomic responses and contributing to the higher mortality observed. Overall, the results highlight the sensitivity of honeybee metabolism to paraquat exposure and provide insight into the metabolic pathways affected by this herbicide. Under field conditions, honeybees may encounter paraquat through both direct exposure during application and indirect exposure through environmental residues. Therefore, understanding the metabolic consequences of different exposure levels may contribute to improved assessment of the ecological risks associated with paraquat contamination.

## 5. Conclusions

This study provided the first comprehensive assessment of the paraquat-induced effects on both upstream (gut microbiome) and downstream (metabolomic) biological processes in *A. mellifera*. Paraquat exposure impaired honeybee health, as evidenced by reduced sucrose consumption and increased mortality, with the LD_50_ treatment resulting in the highest mortality. Although paraquat exposure did not significantly alter the overall gut microbiome composition, both exposures might be associated with modified microbial correlation patterns. In contrast, metabolomic profiling revealed dose-dependent responses, indicating that paraquat primarily disrupts the host metabolic regulation. Both LD_25_ and LD_50_ exposures significantly perturbed the central metabolic pathways, affecting detoxification, antioxidant defense, and energy production. The LD_25_ exposure induced broad metabolic adjustments involving carbohydrate, energy, amino acid, nucleotide, and stress-associated metabolisms, which may represent adaptive responses, whereas LD_50_ also affected carbohydrate and energy metabolism but was associated with metabolic disruption and higher mortality.

Collectively, these findings demonstrate that paraquat induces systemic metabolic reprogramming in *A. mellifera* and is associated with changes in microbial networks, even in the absence of significant shifts in the overall gut microbiome composition. The results indicate that paraquat poses a risk to pollinator performance and may inform beekeeping and herbicide management practices, such as reducing paraquat applications in areas frequented by honeybees and implementing careful herbicide management to protect pollinator populations. Furthermore, we highlight the importance of considering both sublethal and toxic dose effects when evaluating herbicide risks to pollinators and support the use of integrated ‘omics’ approaches to better understand paraquat-induced stress responses. As, in this study, sampling at a single time point following acute exposure may have limited the detection of delayed microbiome restructuring and long-term metabolic responses, future studies incorporating chronic exposure are needed to provide further insight into paraquat’s effects on honeybee health and its long-term side effects associated with field environments.

## Data Availability

The raw sequencing data in this study are available in the National Center for Biotechnology Information (NCBI) under the BioProject accession number PRJNA1310163. The metabolomics data in this study are available from the corresponding author upon reasonable request.

## Supplementary Materials

The following supporting information can be downloaded at https://www.mdpi.com/article/10.3390/insects17060632/s1, Figure S1: Survival curve; Figure S2: The rarefaction curve; Table S1: Table of pairwise comparisons using the log-rank test with the Bonferroni method for survival rate observation; Table S2: Summary of bacterial amplicon sequence variants (ASVs) identified through QIIME2 analysis; Table S3: Table of the relative abundance (%) of gut bacterial taxa across treatment groups; Table S4: The difference in gut bacteria composition between the paraquat treatment groups compared to the control group; Table S5: The difference in gut bacteria composition between the two paraquat treatment groups; Table S6: The alpha diversity of the bacterial microbiome; Table S7: Detected metabolites in the gut of *Apis mellifera* in all samples; Table S8: The significant metabolites in the sublethal paraquat exposure group (LD_25_) compared with the control group; Table S9: The significant metabolites in the toxic paraquat exposure group (LD_50_) compared with the control group; Table S10: The significant metabolites in the toxic paraquat exposure group (LD_50_) compared with the sublethal paraquat exposure group (LD_25_); Table S11: Significant differences in metabolite abundance among groups; Table S12: The top 10 pathways identified by pathway analysis that were disturbed by sublethal paraquat exposure group (LD_25_) compared with the control group; Table S13: The top 10 pathways identified by pathway analysis that were disturbed by toxic paraquat exposure group (LD_50_) compared with the control group; Table S14: The top 10 pathways identified by pathway analysis that were disturbed by toxic paraquat exposure group (LD_50_) compared with the sublethal paraquat exposure group (LD_25_).
