# Supplementary material for: Metabolic and Gut Microbiome Responses to Paraquat Exposure in Apis mellifera Under Laboratory Conditions
Source: Insects. 2026 Jun 15;17(6):632. doi: 10.3390/insects17060632 (PMC13299595; doi:10.3390/insects17060632)
Supplement: Supplementary file 1 [file insects-17-00632-s001.zip › insects-4260832-supplementary.pdf]

Supplementary

Figure S1. Survival curve of *Apis mellifera* among groups.

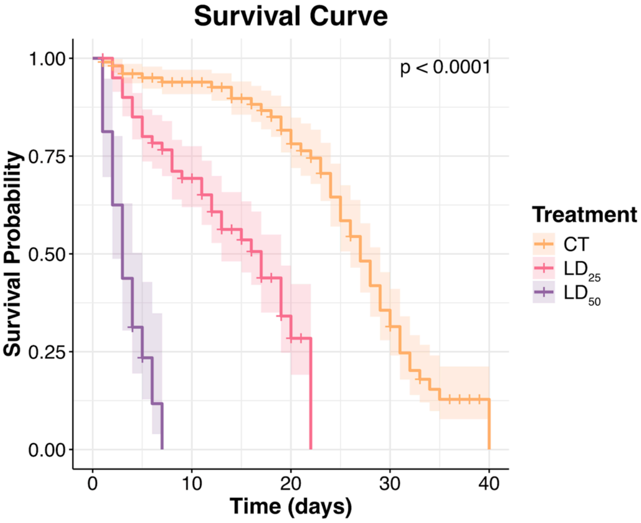

Figure S2. The rarefaction curve.

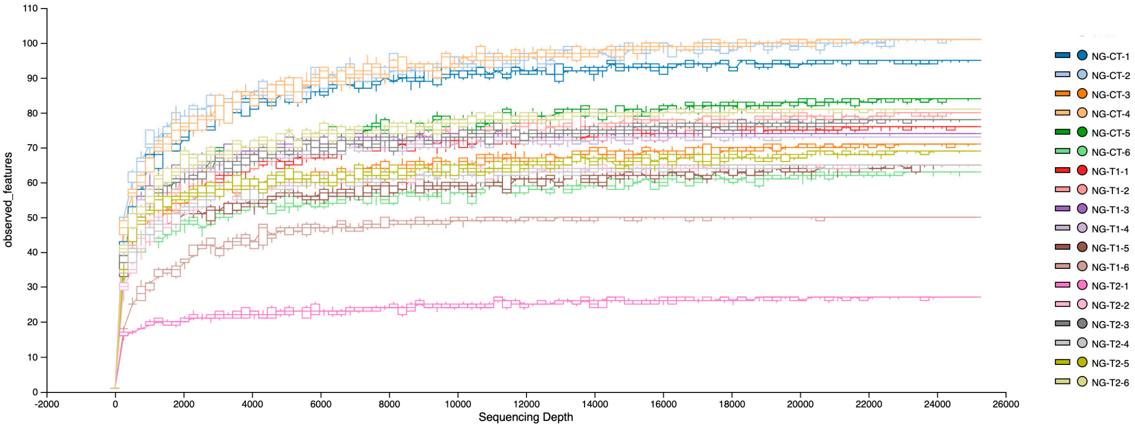

Table S1. Table of pairwise comparisons using the Log-Rank test with the Bonferroni method for survival rate observation.

**Treatment**	**Value**	
	Control (CT)	Sublethal dose (LD_{25})
Sublethal dose (LD_{25})	1.5e-09	-
Toxic dose (LD_{50})	< 2e-16	1.7e-09

Table S2. Summary of bacterial amplicon sequence variants (ASVs) identified through QIIME2 analysis, including demultiplexed sequences data, filtered data, and rarefaction data.

**Demultiplexed sequence counts summary from QIIME2 (The raw bacteria ASVs)**		
	Forward reads	Reverse reads
Minimum	46,479	46,479
Median	58,651.5	58,651.5
Mean	57,851.056	57,851.056
Maximum	69,927	69,927
Total	1,041,319	1,041,319
**Table summary after filtering, quality cut-off, denoising, and removing singletons**		
Metric		Sample
Number of samples		18
Number of features		376
Total frequency		608,663
**Frequency per sample after filtering, quality cut-off, denoising, and removing singletons**		
		Frequency
Minimum frequency		25,164
1st quartile		29,754.75
Median frequency		34,387.5
3rd quartile		37,205.75
Maximum frequency		41,080
Mean frequency		33,814.61
**Table summary after rarefaction (The rarefaction curve ASVs)**		
Summary Statistic		Value
Number of samples		18
Number of features		369
Total frequency		452,952
**Frequency per sample after rarefaction (The rarefaction curve ASVs)**		
		Frequency
Minimum frequency		25,164
1st quartile		25,164
Median frequency		25,164
3rd quartile		25,164
Maximum frequency		25,164
Mean frequency		25,164

Table S3. Table of the relative abundance (%) of gut bacterial taxa across treatment groups (control-CT, paraquat at a sublethal dose-LD_{25}, and paraquat at a toxic dose-LD_{50}) based on QIIME2 analysis, including classification at the phylum, class, order, family, and genus levels.

Phyla	CT	LD_{25}	LD_{50}	Average
Bacteroidota	0.197	0.069	0.007	0.091
Actinobacteriota	17.680	17.304	22.522	19.169
Proteobacteria	25.960	32.227	18.327	25.505
Firmicutes	56.158	50.399	59.141	55.233

Others	0.005	0.002	0.002	0.003
**Classes**	**CT**	**LD_{25}**	**LD_{50}**	**Average**
Clostridia	0.007	0.028	0.023	0.019
Bacteroidia	0.197	0.069	0.007	0.091
Alphaproteobacteria	5.116	10.473	2.699	6.096
Actinobacteria	17.680	17.304	22.522	19.169
Gammaproteobacteria	20.843	21.753	15.628	19.408
Bacilli	56.151	50.370	59.118	55.213
Others	0.006	0.003	0.002	0.004
**Orders**	**CT**	**LD_{25}**	**LD_{50}**	**Average**
Pseudomonadales	0.001	0.000	0.000	0.000
Bacillales	0.000	0.000	0.017	0.006
Lachnospirales	0.007	0.028	0.019	0.018
Flavobacteriales	0.197	0.069	0.007	0.091
Rhizobiales	0.021	0.034	2.188	0.748
Enterobacterales	7.583	1.856	5.396	4.945
Acetobacterales	5.096	10.439	0.511	5.349
Burkholderiales	13.259	19.893	10.232	14.462
Bifidobacteriales	17.680	17.304	22.522	19.169
Lactobacillales	56.151	50.370	59.094	55.205
Others	0.006	0.006	0.014	0.009
**Families**	**CT**	**LD_{25}**	**LD_{50}**	**Average**
Moraxellaceae	0.001	0.000	0.000	0.000
Streptococcaceae	0.000	0.000	0.001	0.000
Bacillaceae	0.000	0.000	0.017	0.006
Lachnospiraceae	0.007	0.028	0.019	0.018
Weeksellaceae	0.197	0.069	0.007	0.091
Enterococcaceae	0.043	0.482	0.278	0.268
Erwiniaceae	0.000	0.032	0.817	0.283
Enterobacteriaceae	0.793	0.352	0.701	0.616
Rhizobiaceae	0.021	0.034	2.188	0.748
Orbaceae	4.795	1.463	3.871	3.376
Acetobacteraceae	5.096	10.439	0.511	5.349
Neisseriaceae	13.259	19.893	10.232	14.462
Bifidobacteriaceae	17.680	17.304	22.522	19.169
Lactobacillaceae	56.108	49.887	58.814	54.937
Others	2.000	0.015	0.021	0.679
**Genera**	**CT**	**LD_{25}**	**LD_{50}**	**Average**
Frischella	0.903	0.717	0.043	0.554
Gilliamella	3.891	0.741	3.790	2.808
Commensalibacter	4.932	10.397	0.461	5.263
Bombilactobacillus	8.551	7.151	7.052	7.585
Snodgrassella	13.197	19.806	10.216	14.406
Bifidobacterium	17.670	17.304	22.518	19.164

Lactobacillus	47.277	42.236	51.540	47.018
Others	3.579	1.648	4.379	3.202

Table S4. The difference in gut bacteria composition between the paraquat treatment groups compared to the control group. The model includes p-values, coefficient values, standard errors, and false discovery rates (FDR).

Feature	Value	coef	stderr	p-value	FDR
Lactobacillus	Sublethal dose (LD_{25})	-0.062	0.054	0.264	0.694
Lactobacillus	Toxic dose (LD_{50})	0.049	0.054	0.379	0.694
Bifidobacterium	Sublethal dose (LD_{25})	-0.008	0.043	0.860	0.926
Bifidobacterium	Toxic dose (LD_{50})	0.053	0.043	0.239	0.694
Snodgrassella	Sublethal dose (LD_{25})	0.064	0.082	0.446	0.694
Snodgrassella	Toxic dose (LD_{50})	-0.031	0.082	0.710	0.904
Bombilactobacillus	Sublethal dose (LD_{25})	-0.016	0.015	0.313	0.694
Bombilactobacillus	Toxic dose (LD_{50})	-0.014	0.015	0.362	0.694
Commensalibacter	Sublethal dose (LD_{25})	0.056	0.069	0.429	0.694
Commensalibacter	Toxic dose (LD_{50})	-0.047	0.069	0.506	0.708
Gilliamella	Sublethal dose (LD_{25})	-0.032	0.035	0.369	0.694
Gilliamella	Toxic dose (LD_{50})	0.0001	0.035	0.998	0.998
Frischella	Sublethal dose (LD_{25})	-0.002	0.007	0.789	0.920
Frischella	Toxic dose (LD_{50})	-0.009	0.007	0.232	0.694

Table S5. The difference in gut bacteria composition between the two paraquat treatment groups (LD_{25} compared to LD_{50}). The model includes p-values, coefficient values, standard errors, and false discovery rates (FDR).

Feature	Value	coef	stderr	p-value	FDR
Lactobacillus	Toxic dose (LD_{50})	0.111	0.054	0.056	0.568
Bifidobacterium	Toxic dose (LD_{50})	0.060	0.043	0.180	0.568
Snodgrassella	Toxic dose (LD_{50})	-0.095	0.082	0.264	0.568
Bombilactobacillus	Toxic dose (LD_{50})	0.002	0.015	0.919	0.919
Commensalibacter	Toxic dose (LD_{50})	-0.103	0.069	0.156	0.568
Gilliamella	Toxic dose (LD_{50})	0.032	0.035	0.371	0.568
Frischella	Toxic dose (LD_{50})	-0.007	0.007	0.346	0.568

Table S6. The alpha diversity of the bacterial microbiome in each treatment group with three metrics in form of mean and standard deviation (Mean ± SD).

Treatment	Diversity metrics		
	Shannon	Simpson	Observed richness
Control (CT)	3.10 ± 0.26	0.92 ± 0.02	85.83 ± 16.06
Sublethal dose (LD_{25})	2.67 ± 0.52	0.86 ± 0.08	64.71 ± 19.40
Toxic dose (LD_{50})	2.72 ± 0.46	0.88 ± 0.06	69.67 ± 10.82

Table S7. Detected metabolites in the gut of *Apis mellifera* in all samples.

Classification	No.	Metabolites
**Benzenoids**		
Methoxyphenols	1	3-Methoxytyramine
Biphenyls and derivatives	2	Adenylsuccinic acid
**Nucleosides, nucleotides, and analogues**		
	3	N6-Methyladenosine
Purine deoxyribonucleotides	4	8-OXO-dGTP
	5	8-OXO-dGDP
Purine 2'-deoxyribonucleosides	6	Deoxyguanosine
Purine ribonucleotides	7	Inosine monophosphate
**Lipids and lipid-like molecules**		
Cholestane steroids	8	Cholesterol
Triradylcglycerols	9	Triglyceride
Fatty acid esters	10	L-acetylcarnitine
	11	2-Hydroxyisovaleric acid
Fatty acids and conjugates	12	Mevalonic acid
	13	Isovaleric acid
**Organoheterocyclic compounds**		
Imidazolines	14	N-methylhydantoin
Tryptamines and derivatives	15	Serotonin
Pyrimidines and pyrimidine derivatives	16	5-Formylcytosine
	17	5-Hydroxymethyl-4-methyluracil
	18	Dihydroneopterin triphosphate
Pterins and derivatives	19	7,8-Dihydroneopterin
	20	Sepiapterin
**Organic oxygen compounds**		
Alcohols and polyols	21	(S)-Propane-1,2-diol
	22	Glycerol
Carbohydrates and carbohydrate conjugates	23	Deoxyribose 5-phosphate
	24	D-threitol
	25	N-acetylneuraminic acid
	26	Fructosamine
**Organic nitrogen compounds**		
Amines	27	Cadaverine
	28	dimethylamine

	29	L-carnitine
Quaternary ammonium salts	30	Acetylcholine
	31	Choline
**Organic acids and derivatives**		
Phosphate esters	32	Phosphoenolpyruvic acid
Carboxylic acids	33	Acetic acid
Dicarboxylic acids and derivatives	34	Succinic acid
Tricarboxylic acids and derivatives	35	Isocitric acid
Alpha hydroxy acids and derivatives	36	2-Hydroxybutyric acid
Alpha-keto acids and derivatives	37	Pyruvic acid
Gamma-keto acids and derivatives	38	Alpha-ketoglutaric acid
	39	4-Hydroxy-2-oxoglutaric acid
Short-chain keto acids and derivatives	40	Acetoacetic acid
	41	Oxaloacetic acid
	42	Ketoleucine
Medium-chain keto acids and derivatives	43	Maleylacetoacetic acid
Amino acids, peptides, and analogues	44	N6-carboxymethyllysine
	45	Homo-L-arginine
	46	homolanthionine
	47	L-methionine
	48	L-glutamic acid
	49	N-methyl-D-aspartic acid
	50	phenylalanine
	51	L-cystathionine
	52	L-Arginine
	53	4-hydroxyproline
	54	glycine
	55	Ophthalmic acid
	56	L-isoleucine
	57	L-Alanine
	58	Citrulline
	59	5-aminolevulinic acid
	60	L-serine
	61	L-cystine
	62	5-Hydroxylysine
	63	L-cysteine

Table S8. The significant metabolites in the sublethal paraquat exposure group (LD_{25}) compared with the control group.

Metabolites	FC	log_2(FC)	raw.pval	$-log_{10}$(p)
Oxaloacetic acid	2.4301	1.281	0.00095044	3.0221
4-Hydroxy-2-oxoglutaric acid	0.58569	-0.7718	0.0033996	2.4686
Glycine	3.8513	1.9454	0.0038245	2.4174
N6-Carboxymethyllysine	0.56079	-0.83448	0.0038245	2.4174
Phenylalanine	1.5137	0.59807	0.0038245	2.4174
Phosphoenolpyruvic acid	0.45667	-1.1308	0.0053922	2.2682
Acetic acid	1.5977	0.67596	0.0053922	2.2682
Mevalonic acid	2.0962	1.0678	0.0060271	2.2199
(S)-propane-1,2-diol	4.2099	2.0738	0.0067263	2.1722
Inosine monophosphate	0.62904	-0.66877	0.018588	1.7308
Cadaverine	1.5677	0.64863	0.044857	1.3482

Table S9. The significant metabolites in the toxic paraquat exposure group (LD_{50}) compared with the control group.

Metabolites	FC	log_2(FC)	raw.pval	$-log_{10}$(p)
7,8-Dihydroneopterin	3.5509	1.8282	0.0092645	2.0332
Oxaloacetic acid	2.1417	1.0987	0.015341	1.8141
(S)-propane-1,2-diol	2.8678	1.52	0.016898	1.7722

Table S10. The significant metabolites in the toxic paraquat exposure group (LD_{50}) compared with the sublethal paraquat exposure group (LD_{25}).

Metabolites	FC	log_2(FC)	raw.pval	$-log_{10}$(p)
4-Hydroxy-2-oxoglutaric acid	1.8932	0.92081	0.037114	1.4305
Phosphoenolpyruvic acid	3.1642	1.6619	0.047085	1.3271

Table S11. Significant differences in metabolite abundance among groups. Group differences were assessed using Mann-Whitney *U* tests for pairwise comparisons ($p < 0.05$).

Metabolites	raw.pval		
	CT-LD_{25}	CT-LD_{50}	LD_{25}-LD_{50}
Oxaloacetic acid	0.0009504	0.01534	0.696
4-Hydroxy-2-oxoglutaric acid	0.0034	0.1837	0.03711
Glycine	0.003824	0.06654	0.8636
N6-Carboxymethyllysine	0.003824	0.1837	0.355
Phenylalanine	0.003824	0.006027	0.7905
Phosphoenolpyruvic acid	0.005392	0.06164	0.04708

Acetic acid	0.005392	0.01138	0.5212
Mevalonic acid	0.006027	0.07727	0.9378
(S)-propane-1,2-diol	0.006726	0.0169	0.2788
Inosine monophosphate	0.01859	0.2188	0.09706
Cadaverine	0.04486	0.2097	0.4064
7,8-Dihydroneopterin	0.07727	0.009265	0.9378

Table S12. The top 10 pathways identified by pathway analysis that were disturbed by sublethal paraquat exposure group (LD_{25}) compared with the control group.

Pathways	Raw p	$-\log_{10}(p)$	Holm adjust	FDR	Impact
Glyoxylate and dicarboxylate metabolism	0.00044792	3.3488	0.016125	0.016125	0.16895
D-Amino acid metabolism	0.0010678	2.9715	0.037372	0.016543	0
Glycolysis or Gluconeogenesis	0.0019245	2.7157	0.065433	0.016543	0.22601
Pyruvate metabolism	0.0019245	2.7157	0.065433	0.016543	0.25001
Terpenoid backbone biosynthesis	0.0022976	2.6387	0.073524	0.016543	0.11392
Glutathione metabolism	0.0069899	2.1555	0.21669	0.04194	0.13766
Folate biosynthesis	0.010442	1.9812	0.31327	0.053703	0.1405
Propanoate metabolism	0.015906	1.7984	0.46127	0.071576	0
Alanine, aspartate and glutamate metabolism	0.019167	1.7174	0.53667	0.076503	0.45271
Porphyrin metabolism	0.021251	1.6726	0.57377	0.076503	0.02795

Table S13. The top 10 pathways identified by pathway analysis that were disturbed by toxic paraquat exposure group (LD_{50}) compared with the control group.

Pathways	Raw p	$-\log_{10}(p)$	Holm adjust	FDR	Impact
Pyruvate metabolism	0.002886	2.5397	0.1039	0.039052	0.25001
Glycolysis or Gluconeogenesis	0.002886	2.5397	0.1039	0.039052	0.22601
Lipoic acid metabolism	0.0037573	2.4251	0.12775	0.039052	0.00189
Glyoxylate and dicarboxylate metabolism	0.0043391	2.3626	0.14319	0.039052	0.16895
D-Amino acid metabolism	0.0057453	2.2407	0.18385	0.041366	0
Alanine, aspartate and glutamate metabolism	0.0071651	2.1448	0.22212	0.042991	0.45271
Terpenoid backbone biosynthesis	0.019698	1.7056	0.59095	0.099966	0.11392
Glutathione metabolism	0.024625	1.6086	0.71412	0.099966	0.13766
Citrate cycle (TCA cycle)	0.024992	1.6022	0.71412	0.099966	0.30043
Porphyrin metabolism	0.028956	1.5383	0.78181	0.10424	0.02795

Table S14. The top 10 pathways identified by pathway analysis that were disturbed by toxic paraquat exposure group (LD_{50}) compared with the sublethal paraquat exposure group (LD_{25}).

Pathways	Raw p	$-log_{10}(p)$	Holm adjust	FDR	Impact
Purine metabolism	0.19962	0.69981	1	0.91398	0.12244
Tyrosine metabolism	0.21401	0.66956	1	0.91398	0.15
Propanoate metabolism	0.22159	0.65445	1	0.91398	0
Glycerophospholipid metabolism	0.23795	0.62351	1	0.91398	0.02771
Lysine degradation	0.35951	0.44429	1	0.91398	0
Valine, leucine and isoleucine biosynthesis	0.37752	0.42306	1	0.91398	0
Sphingolipid metabolism	0.39365	0.40489	1	0.91398	0
Steroid biosynthesis	0.41021	0.387	1	0.91398	0
Insect hormone biosynthesis	0.41021	0.387	1	0.91398	0
Valine, leucine and isoleucine degradation	0.42358	0.37307	1	0.91398	0.01112
